# Fluid dynamic simulations at the interface of the blue-green sharpshooter functional foregut and grapevine xylem sap with implications for transmission of *Xylella fastidiosa*

**DOI:** 10.1371/journal.pone.0265762

**Published:** 2022-03-22

**Authors:** Ian M. Marcus, Daniel White, Elaine A. Backus, Sharon L. Walker, M. Caroline Roper

**Affiliations:** 1 Drexel University, Department of Civil, Architectural, and Environmental Engineering, Philadelphia, PA, United States of America; 2 University of California, Riverside, Department of Chemical and Environmental Engineering, Riverside, CA, United States of America; 3 USDA Agricultural Research Service, San Joaquin Valley Agricultural Sciences Center, Parlier, CA, United States of America; 4 University of California, Riverside, Department of Microbiology and Plant Pathology, Riverside, CA, United States of America; Tongji University, CHINA

## Abstract

*Xylella fastidiosa* is a multi-continental, lethal, plant pathogenic bacterium that is transmitted by sharpshooter leafhoppers (Insecta: Hemiptera: Cicadellidae: Cicadellinae) and adult spittlebugs (Hemiptera: Aphrophoridae). The bacterium forms biofilms in plant xylem and the functional foregut of the insect. These biofilms serve as sources of inoculum for insect acquisition and subsequent inoculation to a healthy plant. In this study, 3D fluid dynamic simulations were performed for bidirectional cibarial propulsion of xylem sap through tube-like grapevine xylem and an anatomically accurate model of the functional foregut of the blue-green sharpshooter, *Graphocephala atropunctata*. The analysis supports a model of how fluid dynamics influence *X*. *fastidiosa* transmission. The model supports the hypothesis that *X*. *fastidiosa* inoculation is mostly driven by detachment of bacteria from the foregut due to high-velocity flow during egestion (outward fluid flow from the stylets). Acquisition occurs by fluid dynamics during both egestion and ingestion (fluid uptake through the stylets and swallowing). These simulation results are supported by previously reported *X*. *fastidiosa* colonization patterns in the functional foregut and sharpshooter stylet probing behaviors. The model indicates that xylem vessel diameter influences drag forces imposed on xylem wall-adherent bacteria; thus, vessel diameter may be an important component of the complex transmission process. Results from this study are directly applicable to development of novel grapevine resistance traits via electropenetrographic monitoring of vector acquisition and inoculation behaviors.

## Introduction

*Xylella fastidiosa* [1] (Xanthomonadales: Xanthomonadaceae) is a xylem-dwelling bacterium that can infect over 500 plant taxa, causing economically severe crop diseases such as Pierce’s disease of grapevine, citrus variegated chlorosis, olive quick decline syndrome, and leaf scorches of oleander, almond, and coffee [2]. Pierce’s disease (PD) of grapevines costs California’s grape growers an estimated $56 million per year in lost production and vine replacements [3]. In addition, the state of California has been spending over $100 million per year to control PD for the last 20 years [4], primarily by vector control through biological control, quarantines, and insecticide applications [5].

*X*. *fastidiosa* is spread by specialized xylem-feeding, insect vectors (Hemiptera: Auchenorrhyncha), chiefly leafhoppers (Cicadellidae) in the subfamily Cicadellinae (called sharpshooters), and spittlebugs (Aphrophoridae) [3]. Among the best-studied vectors are the glassy-winged sharpshooter, *Homalodisca vitripennis* (Germar), and the blue-green sharpshooter, *Graphocephala atropunctata* (Signoret) [6, 7]. *Homalodisca vitripennis*, introduced in the late 1980’s, has been the most destructive vector of PD in California in recent decades [8], although the California native *G*. *atropunctata* is also an important vector, among others [9]. *G*. *atropunctata* was used as the model vector for this study because there is a large breadth of information on its anatomy, physiology, behavior, and vector biology [6, 10–14].

Sharpshooters and spittlebugs are adapted to subsist solely on xylem sap, a highly dilute, nutritionally depauperate food. They must consume up to 1,000 times their body weight per day to filter out amino acids and other nutrients using energetically costly stylet probing behaviors [15]. The physical mechanism, as well as physiological and evolutionary consequences of such prodigious consumption, have eluded scientists for many years [16, 17]. One of the best ways to understand the physical mechanism of xylem sap consumption is to model the fluid dynamics of sharpshooter feeding. Such understanding, in turn, is important because these fluid dynamics power transmission of *X*. *fastidiosa*.

Transmission of *X*. *fastidiosa* consists of three steps, namely: 1) acquisition from the xylem of an infected host plant, 2) multiplication and retention within the vector, and 3) inoculation into xylem of a healthy susceptible plant host [18]. Vectors acquire *X*. *fastidiosa* from xylem sap during specific stylet probing/penetration behaviors. After a presently unknown period of time, these planktonic (suspended) bacteria can then attach to the cuticle in the vector’s functional foregut (precibaria and cibaria) and form biofilms. Biofilms are surface-attached microbial communities encased in a self-produced matrix [19]. Bacteria are then inoculated into xylem of healthy plants during subsequent stylet probes.

**Fig 1:**
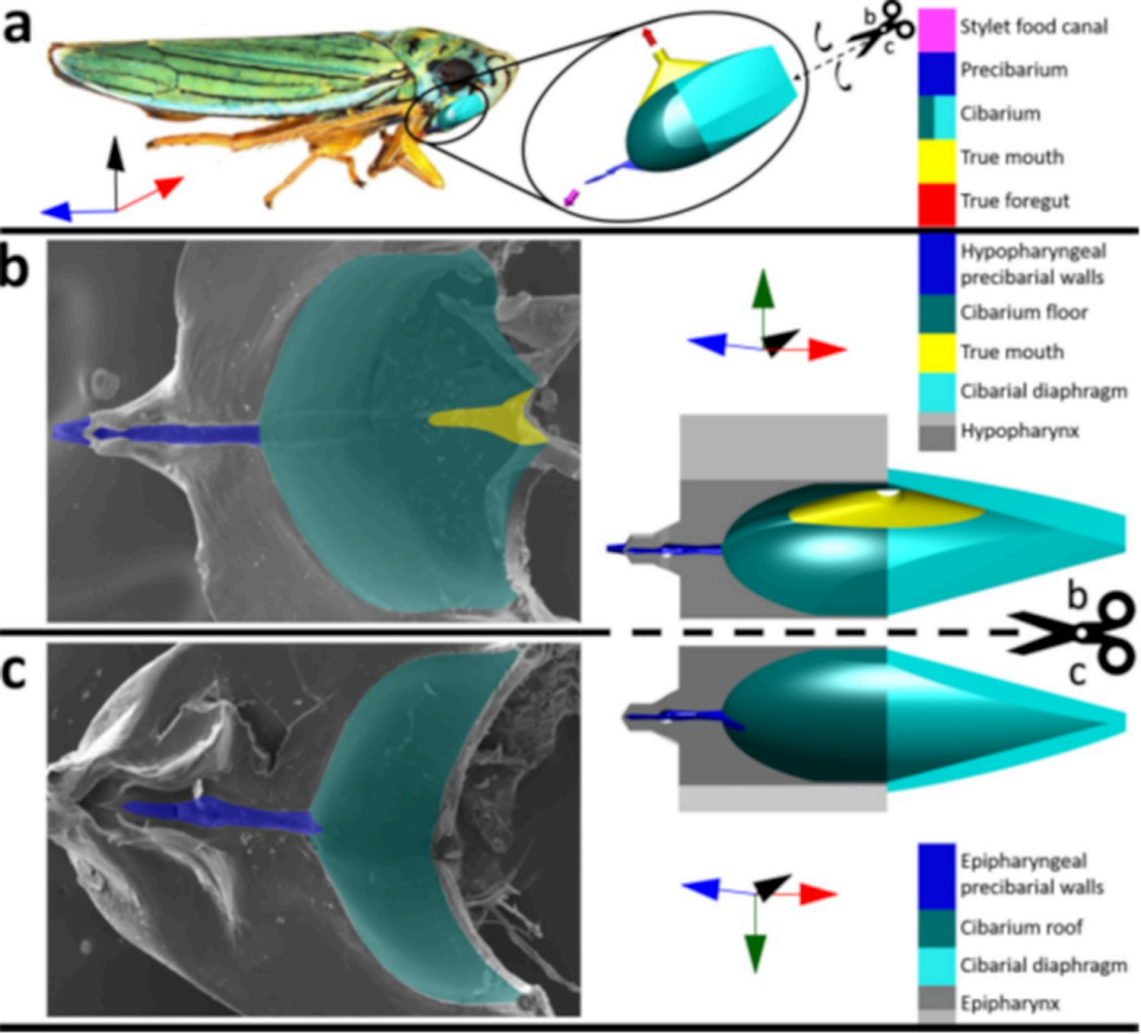
Functional foregut (precibarium and cibarium) of the blue-green sharpshooter, with segments of the 3D model labeled with broad terminology. a) Approximate location/orientation of the functional foregut inside the insect. The 3D model shows an external view of the walls of the precibarium, cibarium, and true mouth, as if the rest of the epipharynx, hypopharynx, and cibarial diaphragm did not exist (convex). b) Hypopharynx electron micrograph (left) and 3D model segment (right, concave, internal) for comparison. The electron micrograph was adapted from a previous publication. c) Epipharynx electron micrograph (left) and 3D model segment (right, concave, internal) for comparison. The coordinate axes indicate anterior → posterior, distal → proximal (for the trough), ventral → dorsal, and left → right. The relationship between the functional foregut and coordinate axes is different for different sharpshooter species. The scissors and dotted lines indicate where the 3D model in subfigure a would be split to obtain the 3D model segments in subfigures b and c. The associated black curved arrows in subfigure a indicate that the two halves of the split 3D model segment would need to be hinged outward to obtain the views in subfigures b and c. The gray 3D parts of subfigures b and c are semi-abstract representations of the epipharynx and hypopharynx that serve for comparison with their corresponding electron microscope images. (Previously published figure from White, et al [20] is reprinted here under the Creative Commons Attribution 4.0 International License).

During *G*. *atropunctata* probing, xylem fluids are taken up by cibarial pumping through the stylet tips, into the stylet food canal, through the precibarium, the cibarium (together, the functional foregut; anatomy shown in Fig 1, [20]), then ingested via swallowing through the true mouth at the proximal end of the cibarium. Technically, ingestion does not occur until the fluid is swallowed. However, for ease of terminology in this paper, we will use the term *ingestion* to refer to all fluid uptake, regardless of whether it is swallowed or not. Conversely, the fluid flow path is reversed during *egestion*, when previously ingested fluids move backwards through the same route and are ejected out the stylet tips. Although sharpshooter ingestion has been documented for many decades, the existence of egestion has been hypothesized as a probing behavior but only recently proven [21, 22].

Acquisition of suspended (planktonic) *X*. *fastidiosa* cells from xylem vessels is thought to occur via ingestion, while inoculation of the same from the foregut is thought to involve egestion. The fluids that carry bacteria during the above behaviors are likely composed of a mixture of xylem sap and saliva [22]. The present study mathematically predicts the physical flow rates by: 1) xylem sap moving through simple, tube-like xylem vessels, a) with and b) without vector feeding, and 2) mixed fluids propelled into or out of vector stylets, a) with and b) without *X*. *fastidiosa* biofilm present in the xylem or vector foregut. Fluid flow rates during ingestion through tube-like models of the precibarium have recently been published [12, 15]. However, fluid flow rates generated by putatively bidirectional cibarial pumping during ingestion or egestion, especially through an anatomically accurate model of the precibarium, have not been produced until our work.

Excluding numerical modeling, as the one presented in the present study, the most rigorous and detailed means of studying stylet probing behaviors of piercing-sucking insects like sharpshooters is via electropenetrogaphy (EPG). In EPG, the insect is made part of an electronic circuit by attaching a thin, gold wire to its dorsum, placing it on a plant, and applying a small (<100 mV) signal to the plant. Specific behaviors like ingestion or egestion performed during stylet probing are transformed into waveforms displayed by computer [23, 24]. After biological meanings of waveforms have been defined, they can be used to identify in real time the performance of specific probing behaviors, which can then be quantified and statistically analyzed to compare among experimental treatments. Sharpshooter waveforms have been extensively studied and defined [25].

For *X*. *fastidiosa* transmission by vectors, the behaviors considered most important are represented by the EPG X wave, a composite waveform that is performed for the first 2–5 min after the stylet tips first penetrate a xylem vessel [16]. The first part of the X wave (XNC) represents a few seconds of egestion with simultaneous salivation; this part alternates with a second part (XC2), consisting of longer (0.5–3 min) events of trial ingestion in each tested vessel [24] (See Section S2.1 in S1 File for further details). Due to the presence of mechanosensilla in the stylets and chemosensilla in the precibarium, the two parts of the X wave phase are interpreted as chemical and mechanical testing of xylem vessels, respectively. Also, successive X waves deposit additional layers of sheath saliva into the vessel wall. Thus, X waves represent sensory behaviors, to test and accept a cell, simultaneous with preparatory behaviors to anchor the stylet tips via sheath salivation [16]. After several X waves are completed, the insect then performs many minutes to hours of sustained ingestion to consummate its meal. Here we will use the term ‘X wave testing” to denote probing behaviors (salivation, egestion, and trial ingestion) represented by alternating parts of the X wave.

We hypothesize that high egestion flow rates can physically detach *X*. *fastidiosa* that may be attached to *G*. *atropunctata*’s precibarium and eject them from the stylet tips, resulting in inoculation [7, 11]. The likelihood of this inoculation mechanism occurring can be evaluated by simulating fluid dynamics in the *G*. *atropunctata* precibarium during egestion and comparing the results with the drag forces required to detach *X*. *fastidiosa*. Current models of flow in the insect functional foregut [11, 12, 15] do not simulate egestion and also lack the geometric fidelity to the complex precibarial anatomy required for nuanced estimation of drag forces acting on attached bacteria. Here, we simulated precibarial fluid dynamics during egestion using a 3D model of the *G*. *atropunctata* precibarium [20] appropriate for nuanced drag force predictions. Precibarial fluid dynamics during ingestion were also simulated to gain understanding about *X*. *fastidiosa* acquisition from infected xylem vessels.

In response to fluid flow rates, *G*. *atropunctata* ingestion and/or egestion must cause fluid drag forces in xylem vessels at the same time as they cause fluid drag forces in the functional foregut. Such drag forces from vector probing may cause detachment of *X*. *fastidiosa* from biofilm on plant xylem walls, a potentially important step in bacterial acquisition. To our knowledge, fluid dynamics that occur in xylem during insect ingestion or egestion have not been previously simulated, nor have the drag forces acting on wall-attached bacteria been calculated. These fluid dynamics would intuitively affect xylem-dwelling biofilms, if the drag forces acting on them are comparable to or greater than those present before the vessels are disturbed by insect feeding (termed *Undisturbed* xylem herein).

Bouda, et al. [26] made estimations of undisturbed xylem flow rates in one-year old grapevine using the Hagen-Poiseuille equation. This equation predicts total flow rates in cylindrical pipes based on the viscosity of the liquid, the pressure gradient along the pipe length, and the radius of the pipe. Based on their analysis, simulations of xylem flow with a uniform pressure gradient across multiple, cylindrical vessel elements (linked end-to-end to form pipe-like xylem vessels) provided representative predictions in narrow xylem vessels and overpredicted flowrates in wide vessels by an average of 23%. Thus, simulations of xylem flow in grapevine using the Hagen-Poiseuille equation and the average xylem pressure gradient should allow order of magnitude predictions of undisturbed xylem flowrates. However, these flowrates would not provide the resolution required to estimate drag forces acting on surface-attached bacteria, which require estimations of fluid shear rates.

Instead, shear rates can be better-predicted in simulations based on the Navier Stokes equations. These are equations that can be used to derive the Hagen-Poiseuille equation. Similar to the Hagen-Poiseuille equation, the Navier Stokes equations require input of the axial pressure gradient to predict flowrates. Previous simulations of xylem flow using the Navier Stokes equations have used the water potential gradient in place of the pressure gradient, though water potential gradients were not based on direct experimental measurements [27]. Water potential gradient is a more complete measure of the driving force of xylem flow (pressure potential due to evapotranspiraton, combined with osmotic potential and matrix potential) than the pressure gradient alone. The water potential gradient has been reported as 0.03 MPa/m for both well-watered and water-stressed grapevines [28]. We used this reported gradient together with the Navier Stokes equations to predict drag forces acting on surface-attached bacteria in undisturbed grapevine xylem.

In this study, we used novel fluid dynamics simulations to investigate the role of physical forces generated by the *G*. *atropunctata* cibarium on fluid movements through the precibarium, then into and out of stylets. We also considered implications of our findings in relation to *X*. *fastidiosa* transmission (both acquisition and inoculation). Grapevine xylem simulations predict drag forces can act on bacteria in the xylem during insect ingestion and egestion during undisturbed conditions. *G*. *atropunctata* functional foregut simulations predict fine-scale drag forces during ingestion and egestion. Drag force profiles for blue-green sharpshooter were compared with condensed versions of previously reported spatial colonization patterns of *X*. *fastidiosa* biofilm in the precibarium [6]. Simulation and comparison results support new mechanistic insights relevant to pathogen transmission. Our model can serve as a platform to generate future, testable hypotheses regarding the mechanisms underlying transmission, and whether these could be disrupted to mitigate diseases caused by *X*. *fastidiosa*.

## Results

### Simulations of cibarial flowrates

For our model calculations, we assumed that the cibarial diaphragm would be uplifted by the cibarial muscles to the maximum allowable degree (that is, full length of the diaphragm, Fig 1) for each pump, thus powering the maximal flow rates for fluid exiting the cibarium, as well as drag forces allowable. Although maximum diaphragm uplift may seem anatomically unlikely, our simulations are based on the mean flowrates averaged across all cross-sectional areas within the cibarium (S5B Fig in S1 File). Because we have made order-of-magnitude predictions of mean flow rates, any cibarial filling assumptions between 10% and 100% are not expected to change calculated drag forces. As a result, our simulations likely underpredict the flowrates that impact bacterial detachment in the prebarium and xylem. EPG studies have shown that amount of uplift of the diaphragm is highly variable and controllable by the insect, as documented via the amplitude of an egestion spike (one expulsion) or ingestion plateau (one “gulp” and swallow) [25]. Typically, the egestion spike is much larger than the ingestion plateau. Taking all of the above into consideration, we do not expect that our calculated mean drag forces are overpredicted.

**Fig 2:**
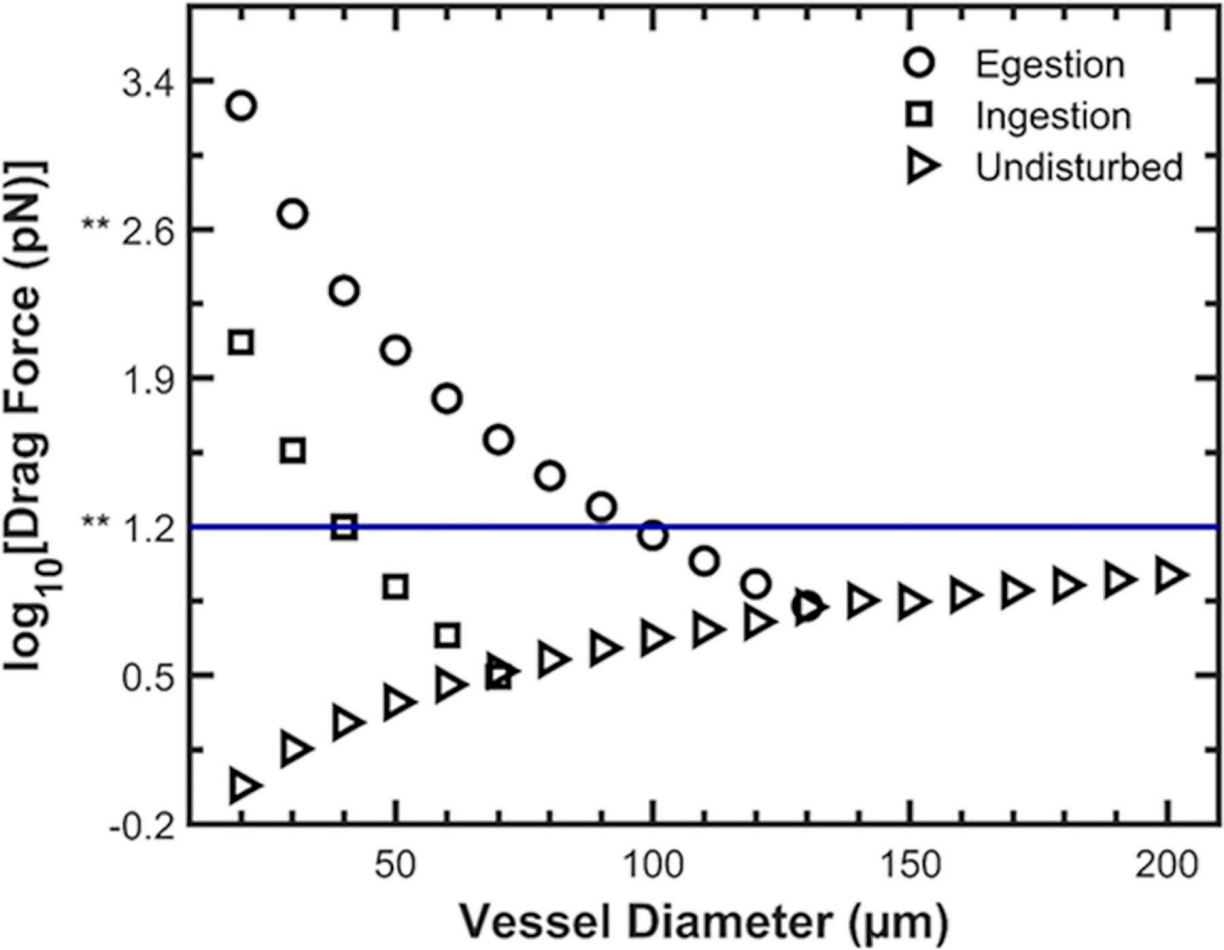
Simulated drag forces acting on individual, surface-attached bacteria in grapevine xylem vessels. ‘Egestion’ and ‘Ingestion’ indicate simulated fluid flow for insect-xylem interactions during these respective activities. ‘Undisturbed’ indicates simulated undisturbed xylem vessels by modeling the water tension gradient as a pressure gradient. The ** symbols on the y-axis are references to key drag forces in the results of a previous microfluidic study [29]. The blue horizontal line is a visual aid at 10^1.2^ pN (15.85 pN) that represents the drag force where less than 1% of individually surface-attached Xylella fastidiosa cells detached in De la Fuente et al. [29]. When the drag forces in that same study were sequentially increased in our model, over 70% of X. fastidiosa detached by the time the drag forces reached 10^2.6^ pN (443.8 pN) [29].

Calculated mean drag forces from the insect were greater than or equal to those in *Undisturbed* xylem, for vessels smaller than 80 μm and 140 μm during ingestion and egestion, respectively (Fig 2; *Ingestion*, *Egestion*). For vessels smaller than 80 μm, mean predicted drag forces were at least 14 times higher during egestion than during ingestion (see Section S2.1 in S1 File for further explanation).

### Xylem simulations

We simulated fluid dynamics for xylem vessels during *G*. *atropunctata* probing, when ingested and egested fluids flow into and out from (respectively) the insect stylet tips that penetrate a xylem vessel, respectively (Section S2.1 in S1 File).

We simulated fluid dynamics in an undisturbed grapevine xylem vessel, defined herein as xylem that is not being stylet-probed by *G*. *atropunctata* (Fig 2, *Undisturbed*) [27]. Results indicate that both flow rates (S2 Table in S1 File) and drag forces (Fig 2) in *Undisturbed* xylem increase with increasing vessel diameter. The predicted drag forces are not high enough to detach individual bacterial cells attached directly to the vessel wall surface, assuming *X*. *fastidiosa* adhesive forces when adhered to xylem walls are similar to those reported when *X*. *fastidiosa* is adhered to glass in microfluidic studies [29]. However, *Undisturbed* drag forces may be high enough to detach clumps of bacteria attached to one another in later stages of biofilm development because *X*. *fastidiosa* biofilm stiffness decreases over time *in vitro* [30]. Such cellular aggregates can detach from biofilms and move downstream under flow conditions comparable to those simulated in *Undisturbed* xylem (S2 Table in S1 File) [30].

This indicates that the insect’s mean cibarial muscle strength is sufficient to overcome the natural drag forces caused by the upward fluid pull of evapotranspiration and water potential, in xylem vessels smaller than 80 μm. The difference between insect-driven drag forces (*Ingestion*, *Egestion*) and undisturbed xylem drag forces (*Undisturbed*) in vessels of a given diameter decreases with increasing vessel diameter (Fig 2).

### Precibarium Simulations

**Fig 3:**
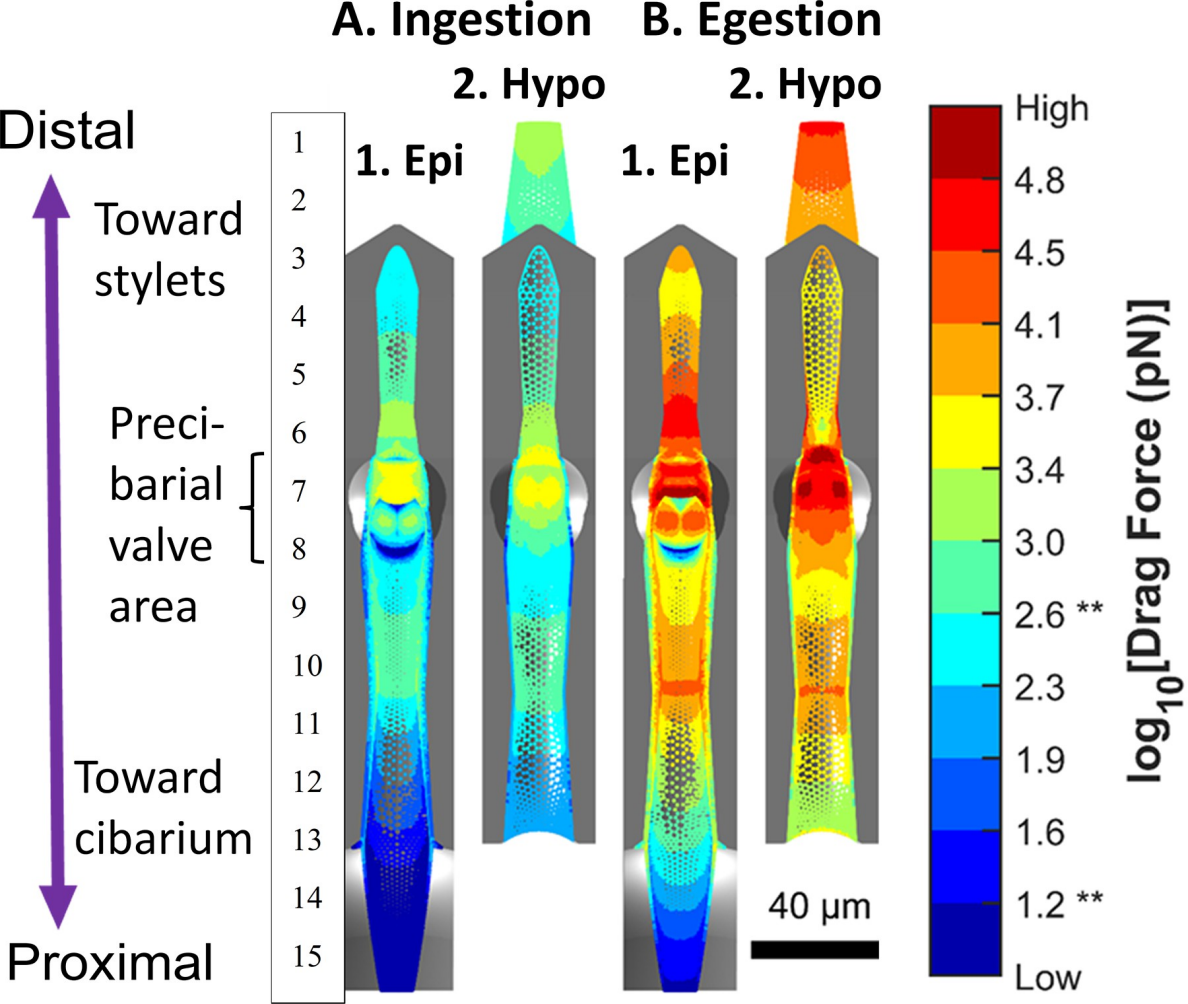
Colormaps of simulated drag forces acting on individually surface-attached bacteria in the *G*. atropunctata precibarium during A. Ingestion and B. Egestion. Colors representing drag forces (guide to the right of colormap) are plotted within gray 3D models of the two halves of the precibarium, the epipharynx (1. Epi) and hypopharynx (2. Hypo), which are similar to previously described 3D models (Fig 1). Numerals to left of colormap represent specific precibarial locations (see text references). During ingestion, drag forces on both halves of the precibarium are generally lower (bluer) than during egestion (yellow to red). The ** symbols on the numerical colormap guide are references to key drag forces in the results of a microfluidic study [29]. 10^1.2^ pN (15.85 pN) is the drag force where less than 1% of individually surface-attached X. fastidiosa cells detached in De la Fuente et al (2007). When the drag forces in that same study were sequentially increased, over 70% of X. fastidiosa detached by the time the drag forces reached 10^2.6^ pN (443.8 pN) [29]. The number labels are present to highlight the location of insect anatomy within the precibarium that are further described in text. See White, et al., [20] for full description of each segment of the precibarium.

We also created fluid dynamic simulations for fluid flowing through, both into and out of, the *G*. *atropunctata* precibarium. Full plots of simulation results can be found in the SI (S1 and S2 Figs in S1 File). Results herein focus on the precibarium due to its relevance for *X*. *fastidiosa* transmission, both acquisition and inoculation [6]. Simulation results are plotted as colormaps within 3D molds [20] that resemble segments of the two halves of the precibarium, the epipharynx and hypopharynx (Fig 3). The colormaps represent the calculated drag force experienced by an individually surface-attached bacterium at a given location.

There are four segments of the precibarium with drag forces under 10^1.2^ pN during both ingestion and egestion (referred to as “low predicted drag forces” herein). All such segments are colored dark blue in Fig 3A (Ingestion) and 3B (Egestion), near the following numbered labels in Fig 3A: the precibarial pit/ring (between labels 6 and 7), the valve shadow (label 7), the basin crease (label 8), and the proximal end of the cibarial epipharyngeal trough (label 15). The drag force 10^1.2^ pN (15.85 pN) is where less than 1% of individually surface-attached *X*. *fastidiosa* cells detached in a microfluidic study [29]. We hypothesize that slow-velocity eddies, otherwise referred to as recirculation zones where the fluid recirculates, would be most likely to form in these locations. Thus, we propose that these areas of low predicted drag forces in the precibarium likely serve as important sites of bacterial colonization (that is, attachment to form biofilms during acquisition).

When drag forces in the same microfluidic study were incrementally increased, over 70% of *X*. *fastidiosa* detached by the time the drag forces reached 10^2.6^ pN (443.8 pN) [29]. In Fig 3, several segments of the precibarium were predicted to experience drag forces more than 1.5 orders of magnitude higher than 10^2.6^ pN during egestion (referred to as “high predicted drag forces” herein). These areas are colored pale green to dark red in Fig 3A (Ingestion) and especially 3.B (Egestion). They include the epipharyngeal trough peak (Fig 3A1 and 3B1, label 7), hypopharyngeal trough peak (3A2 and 3B2, label 7), and the epipharyngeal valve contact patch (3A1 and 3B1, label 6). We propose that these highly sculpted cuticular areas of high predicted drag serve as sources of inoculum during the inoculation process, because egestion drag forces are over 10^1.2^ pN, and thus bacteria can be readily dislodged from these areas.

### Simulations of bacterial colonization patterns in the precibarium

**Fig 4:**
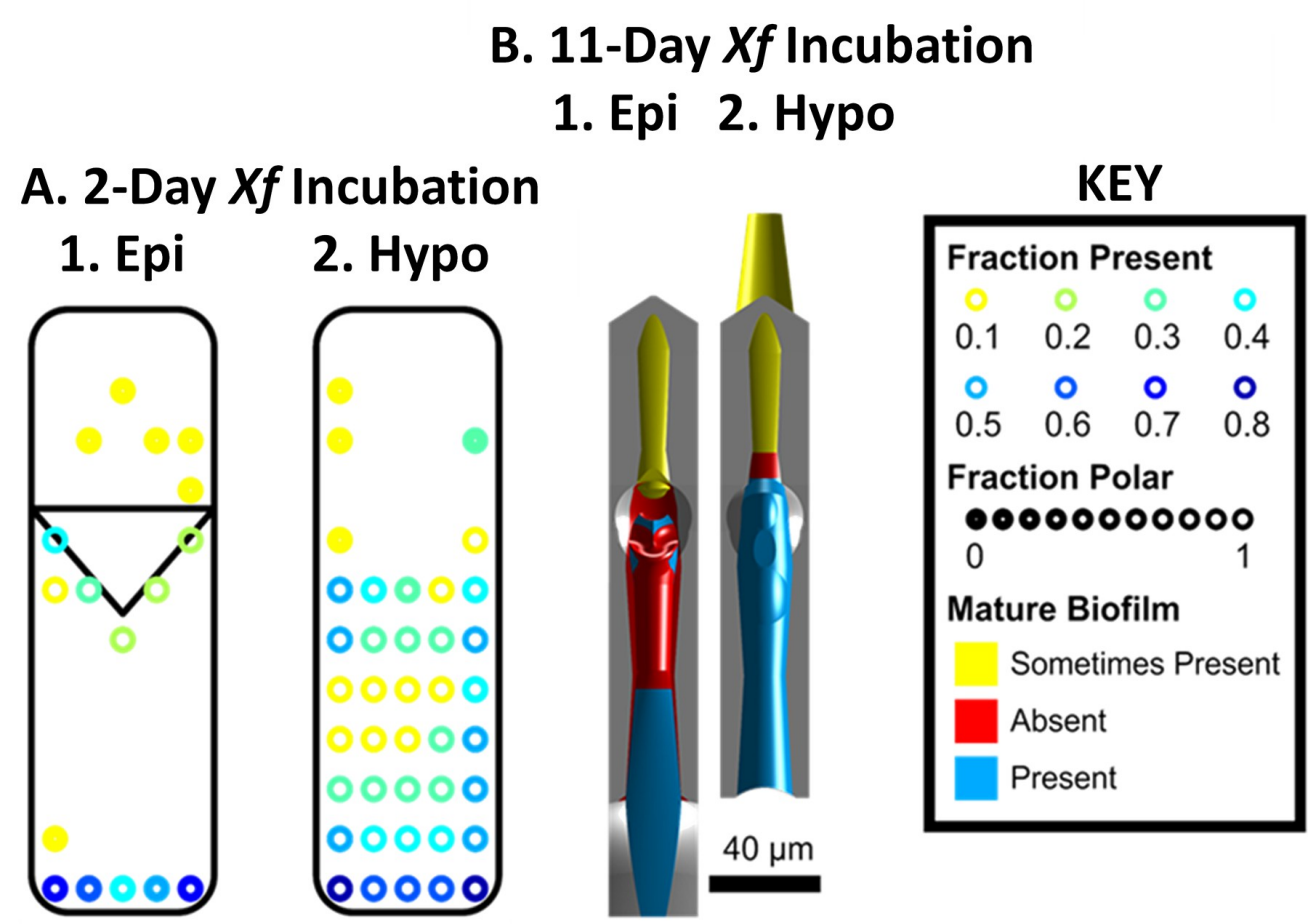
Colonization/biofilm patterns of X. fastidiosa (Xf) in the precibarium of *G*. atropunctata after A. a 2-day incubation period, and B. an approximately 11-day incubation period; 1. Epipharynx (Epi), 2. Hypopharnx (Hypo). A. Subfigures summarize the findings of Almeida and Purcell [6] by combining their 12 published diagrams of epipharynx and hypopharynx colonization. These images are not drawn to scale of the precibarium, and the triangle represents the precibarial valve flap. The color of the circles represents the fraction of published observations of a given location that indicated cells were attached (see KEY). The amount of circle fill represents the fraction of attached cells that were polarly attached. B. Subfigures summarize the location of developed biofilms in the precibarium, as gathered from several published descriptions and published microscope images [6, 12, 13, 32]. Blue represents colonized segments, red represents uncolonized segments, and yellow represents segments that are sometimes colonized.

Almeida and Purcell previously observed patterns of bacterial colonization of *X*. *fastidiosa* in the precibaria of *G*. *atropunctata* after a 2-day incubation period (1-day acquisition access period and a 1-day inoculation period) [6]. Their results were recorded in a collection of 12 diagrams. We condensed these diagrams into a single figure for comparison with drag forces predicted by our simulations (Fig 4A). Similarly, colonization patterns described and observed for developed *X*. *fastidiosa* precibarial biofilms (11-day incubation period) in the literature [6, 12, 13, 31] were condensed onto 3D plots of the precibarium (Fig 4B). These studies were chosen since they display images of mature biofilm within an insect. It should be noted that previous research demonstrating colonization patterns in a dynamic time-course experiment was not used, to keep the model simple [7].

Precibarial colonization patterns support the hypothesis that flowrates during egestion are sufficient to detach bacteria attached to the cuticle. Segments of the precibarium with higher predicted drag forces (Fig 3B) tended to have lower bacterial colonization frequencies after a 2-day incubation period (Fig 4A1 and 4A2). Segments with lower predicted drag forces tended to have higher colonization frequencies. Colonization patterns in the proximal (lower in the figure) two thirds of Fig 4A2 resemble the saddle-like drag forces in Fig 3B2, with less colonization in the middle and more at both ends of the precibarium. The segments of the precibarium that appear to be distal to the precibarial valve, with drag forces greater than 10^3.0^ pN during egestion (Fig 3B1 and 3B2), were not often colonized (Fig 4A1 and 4A2). In contrast, the proximal-most regions of the hypopharynx and epipharynx, with some of the lowest drag forces on the hypopharynx (Fig 3A1 and 3A2), had the highest likelihood of observing attached bacteria, thus high rate of colonization (Fig 4A1 and 4A2). These colonization patterns support our model of the fluid dynamics within the precibarium.

Two of the other precibarial regions with low predicted drag forces were likely colonized during early biofilm formation, well before the end of the 2-day incubation period, also shown in Fig 3A1 and 3B1. They were the valve shadow (label 7) and the precibarial pit/ring (between label 6 and 7), because (in scanning electron micrographs) bacterial cells were observed seeming to spill or grow out from under the valve shadow and out of the entrance of the deep precibarial pit, respectively, after only a short, 2-day incubation period [6]. Cells also were often found inside the precibarial pit (within the ring, between label 6 and 7, Fig 3), though it is not clear whether this included specimens with 2-day incubation periods. In addition, Fig 3A1 indicates bacterial colonization near the edge of the precibarial valve/flap (label 7) [31, 32], which is reminiscent of previously published scanning electron micrographs of developed biofilms that appear to extend under the valve flap [6, 14]. Therefore, three of the four regions with low predicted drag forces were likely colonized during early biofilm formation because their colonization would require time, thus probably started early in biofilm formation.

The fourth region, the basin crease (Fig 3A1 and 3B1; dark blue semi-circle at label 8), remained notably uncolonized when developed, 11-day *X*. *fastidiosa* incubated biofilms were observed in the precibarium (Fig 4B1). The lack of colonization in this region contradicts a simple flow-driven attachment-detachment interpretation, and thus may be due to water hammer. Water hammer is a phenomenon observed in water pipes where a pressure wave travels through the water after a valve is abruptly closed [33]. Cervantes and Backus [13] interpreted results from Dugravot, et al. [34] describing repeating plateaus that represent individual cibarial pumps in EPG recordings of *G*. *atropunctata* ingestion; a small peak at the end of each plateau is thought to represent closing of the precibarial valve just before or instantaneous with swallowing. Their findings indicate that this closure occurs while fluid is continuously flowing into the cibarium during ingestion and, thus, interrupts this process. In this case, quick closure of the valve likely causes water hammer.

Simplistic calculations indicate that instantaneous closure of the precibarial valve during ingestion could increase the pressure distal to the valve by four atmospheres. The resulting pressure force could kick and create current distally, in the distal enclosure formed by both sides of the precibarium (Fig 3A, labels 4 to 5). Additionally, the pressure force may kick the epipharyngeal basin proximally, creating a water current in the basin crease and basin proximal slope (Fig 3A and 3B, labels 6 to 7). In either case, cells could be detached in both directions from the valve as it closes. This hypothesis is supported by observations in other parts shown in Fig 3A1 and 3B1, that is, colonization of the basin triangles (label 9), but lack of colonization between the basin triangles and the epipharyngeal fold (vertical line between labels 7 and 8) [6]. This suggests that the epipharynx folds “horizontally” (in the view of Fig 3) so that the two indicated boundaries approach each other during water hammer, smashing or dislodging cells at the edges of the basin triangle.

Other segments of the precibarium that remained uncolonized include the middle portion and proximal edge of the epipharyngeal trough (Fig 3A1 and 3A2, labels 13 through 15). These are segments with predicted drag forces that are mostly greater than 10^3.0^ pN during egestion (Fig 3B1 and 3B2, labels 12 through 15). Additionally, the distal epipharyngeal basin (Fig 3A1 and 3B1, labels 7 and 8) and the valve contact patch (Fig 3A1 and 3B1, label 6) remained uncolonized (Fig 4); however, these may additionally be due to mechanical rubbing by closure of the precibarial valve [6]. Other segments distal to the precibarial valve were sometimes colonized, but only after pervasive biofilms developed proximal to the preciabrial valve in the precibarium (Fig 4). These segments normally demonstrated predicted drag forces greater than 10^3.0^ pN during egestion (Fig 3B), but drag forces could be reduced when extensive biofilm formed at or above the valve (see below).

Notably, there are two main locations in Fig 4 where higher frequencies of laterally attached bacteria were seen after 2-day incubation periods. Lateral attachment occurs when formerly planktonic (suspended) bacteria first attach to the cuticle. Later, as the cells begin forming their exopolysaccharide (EPS) adhesion material, they pull up and become polarly attached within the mature biofilm [35]. The two aforementioned locations are: 1) distal to the precibarial valve (the distal enclosure) (Fig 3A1 and 3B1, label 3 to 6) and 2) in a proximal segment of the epipharyngeal trough (Fig 3A1 and 3B1, labels 10 to 14). We interpret presence of recently attached bacteria, thus immature biofilm, as demonstrating that drag forces are so severe at these locations that they are usually scrubbed clean. This finding matches that of Backus and Morgan [7], who found that colonization distal to the valve only occurred when pervasive biofilm occurred proximal to the valve, with suggestion of biofilm growth over the valve, presumably blocking its functioning.

## Discussion

The complex transmission process of *X*. *fastidiosa* occurs at the interface of two different aqueous niches, the insect functional foregut and the plant xylem, both under hydrodynamic flow. Our simulations provide high-resolution details of how fluid dynamics (mean flow rates and drag forces) in both of these environments influences and facilitates the comprehensive process of transmission (acquisition, retention, and inoculation). Using this data, we created a model of how fluid dynamics under mean drag forces created by the insect impact bacterial attachment/colonization and detachment from both the xylem vessel wall and the insect functional foregut (precibarium and cibarium) during ingestion and egestion. Ingestion is defined herein as sucking xylem sap (or a mixture of sap and saliva) into stylets and the functional foregut of *G*. *atropunctata*, and egestion is defined as expelling such fluid out from the same.

In the following discussion, our insect and xylem simulation results are used to gain mechanistic insights into *X*. *fastidiosa* acquisition and inoculation, by examining: 1) drag forces in undisturbed xylem vessels and their effect on bacterial attachment/movement, then 2) drag forces caused by ingestion and egestion during *G*. *atropunctata* feeding and their effects on bacterial attachment/movement. EPG has shown that, during X wave testing of a xylem vessel by the insect, events of egestion (with co-occurring salivation; waveform XNC) and trial ingestion (waveform XC2) rapidly alternate with one another within seconds to minutes [11, 16, 24]. Despite this complex temporal dynamic, for ease of understanding, here we will first discuss ingestion and egestion independently, then consider them together, for first acquisition then inoculation.

### Flow within the xylem

Both predicted flow rates and drag forces in undisturbed xylem vessels increase with increasing vessel diameter. Although drag forces are not high enough to detach individual bacterial cells attached directly to the vessel wall surface [29], undisturbed drag forces may be high enough to detach clumps of older, stiffer bacterial aggregates from the biofilm and move downstream [30]. Element-to-element, downstream movement within a pipelike xylem vessel may be more facilitated in wider vessels because the cells remain planktonic and free-floating in the xylem, enabling movement into adjacent xylem elements. Systemic movement in the plant is tightly linked to *X*. *fastidiosa* virulence [26–28].

### Flow into the insect: Acquisition

The simulations in the insect and xylem models indicate that drag forces during ingestion are, as we hypothesized, sufficient to dislodge bacteria from the xylem wall and to draw bacteria into the functional foregut during the ingestion process. Thus, one need not think of already-floating, planktonic (suspended) bacteria in the xylem flow as the only cells susceptible to uptake; attached cells in xylem biofilm likely can be detached by the suction/drag force exerted during *G*. *atropunctata* ingestion. Active detachment of planktonic bacteria from biofilm could increase bacterial concentrations in xylem vessels, allowing more attachment in the precibarium. This is because bacterial deposition rates can be approximated as being proportional to floating bacterial concentration [36]. The timing of bacterial acquisition is discussed further in the SI. Notwithstanding the above discussion about ingestion, our fluid dynamics research cannot evaluate whether watery, enzymatic saliva may also play a role in dislodging attached bacteria.

Our xylem simulations also suggest that vessel diameter can be one factor of many impacting bacterial acquisition by vectors, because drag forces caused by ingestion were predicted to be higher within narrow, more flow-resistant xylem vessels (less than 140 μm in diameter) as compared to wider, less flow-resistant vessels (greater than 140 μm in diameter). This hypothesis is supported by observations that *H*. *vitripennis* exhibits higher bacterial acquisition efficiencies from water-stressed grapevines than from well-irrigated grapevines [37]. Nonetheless, biological interactions among plant, bacteria, and vector are likely to be more complicated than our calculations can fully predict. For example, Krugner and Backus used EPG to compare *H*. *vitripennis* probing behaviors on water-stressed versus well-irrigated plants, either citrus or almond. Insects performed shorter and less frequent events of both trial and sustained ingestion on water-stressed plants, but also less on citrus (regardless of water status) than on almond. Citrus is a less preferred host plant than almond [38]. Future experimentation will be needed to determine whether xylem vessel diameter impacts bacterial acquisition by vectors.

Until the present work, most acquisition was previously linked to sustained ingestion. Egestion has been primarily associated with *X*. *fastidiosa* inoculation rather than acquisition. Notably, herein we hypothesize that egestion drag forces, which are at maximum 14 times higher during egestion than ingestion, are even more likely than ingestion forces to agitate and disperse xylem wall-bound biofilm, especially from narrower vessels. The resulting planktonic bacteria can then be taken up into the foregut when the sap-plus-saliva mixture is subsequently ingested. Thus, powerful egestion probably occurs in tandem with ingestion to achieve bacterial acquisition. Our model supports that there is potential for both acquisition and inoculation in each tested xylem vessel, thus during trial ingestion, not just during sustained ingestion. This action can be relatively rapid; if bacteria are present in the xylem, acquisition likely begins during egestion and is completed during both trial ingestion and sustained ingestion.

### Flow out of the insect: Inoculation

It is clear from the above results and discussion that bacteria can probably transition from an attached state to detached, planktonic state rapidly under the influence of either egestion or ingestion. When planktonic bacteria are ingested and then almost immediately (within a few second to minutes) egested, for example when the stylets move between adjacent xylem vessels in the same plant, or after the insect briefly hops or walks to an adjacent leaf, then there will be essentially no latent period. In such cases, it is presumed that retention is too brief for much attachment to occur, and the still-planktonic bacteria can be egested. In this relatively rare situation (as stated in the original paper [22]), the sharpshooter can be considered a “flying syringe.”

In most circumstances in the field, however, inoculation probably is dominated by detachment rather than a flying syringe mechanism. In that case, our inferred narrative model is supported by the results of Purcell and Finlay, which are consistent with increased *X*. *fastidiosa* inoculation via *G*. *atropunctata* with longer inoculation access periods (IAP) [39]. If inoculation of planktonic bacteria from the fluid held in the functional foregut were a large contributor to transmission, as in the flying syringe model, the predicted transmission rate would still be >20% for a 1-hour IAP. However, the transmission rate (2%) is less than one tenth of that value. In addition, *X*. *fastidiosa* transmission via *G*. *atropunctata* that were tested for different acquisition access periods (AAP) increased with increasing AAP. If transmission occurred solely due to a flying syringe mechanism, the transmission would not be affected by AAP.

We hypothesize that flow-driven detachment is the main cause of *G*. *atropunctata*’s *X*. *fastidiosa* inoculation in grapevine vineyards. Besides the flying syringe hypothesis, it also has been hypothesized that insect saliva plays a biochemical role in detaching bacteria from the functional foregut by degrading biofilm EPS [40, 41]. In contrast, our fluid dynamic simulations predict drag forces in the precibarium much higher than those required to detach *X*. *fastidiosa* from glass in the absence of insect saliva (Fig 2) [29], making egestion necessary and sufficient by itself for inoculation. Thus, biochemical degradation may not be necessary for *X*. *fastidiosa* transmission. Nonetheless, our research cannot exclude a potential role for saliva in bacterial inoculation; only future research can determine it.

Intermittent inoculation could be explained by quiescent periods in the precibarium alternating with high flow periods, such as egestion, causing much of newly attached biofilm growth to detach. Only bacteria that are in low-flow regions or that have a high degree of adhesion tenacity would remain attached to the surface.

While our fluid dynamic simulations are useful for predicting the mechanics underlying *X*. *fastidiosa* transmission, they have limitations, discussed further in the SI, that could be improved in future studies. Nonetheless, our model can be applied to many different experimental systems in future work, to add a valuable fluid dynamics understanding.

### Summary and conclusions

**Fig 5:**
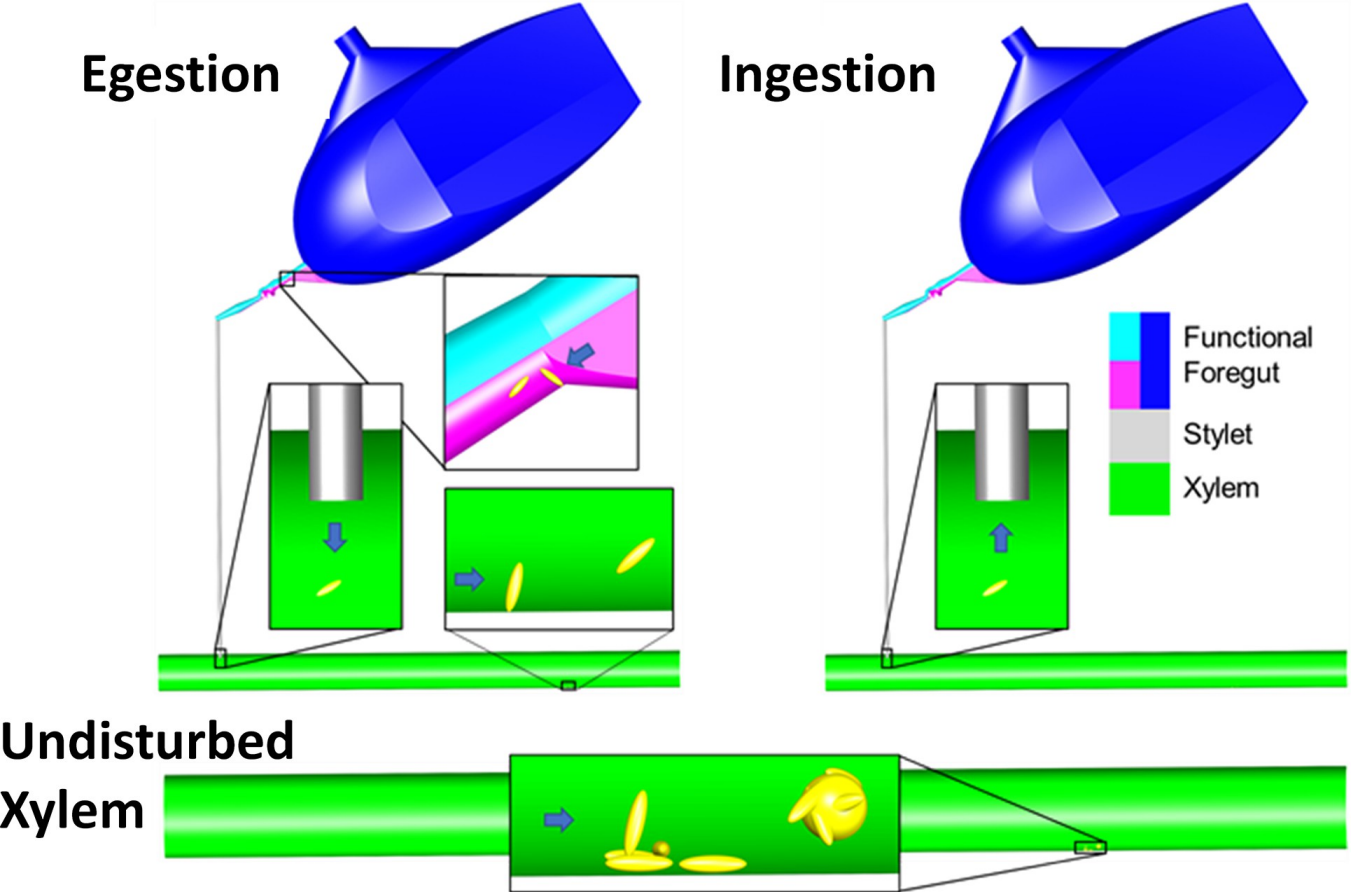
Inferred model of detachment-based transmission and virulence. The model describes the effects of xylem sap flow on surface-attached bacteria during ‘Egestion,’ ‘Ingestion,’ and in ‘Undisturbed Xylem.’ The cibarial diaphragm is probably not fully extended, as shown here, for ingestion, but is likely for very powerful egestion. See text for detailed description. Stylet = stylet bundle, or all four stylets held together to form a tube.

We infer the following narrative based on the preceding analysis (Fig 5). Xylem flow, driven by a water potential gradient [42], causes gradual detachment and spread of *X*. *fastidiosa* in undisturbed xylem. When *G*. *atropunctata* probes grapevine xylem, high ingestion and egestion flowrates can cause xylem-dwelling bacteria to rapidly detach and be pulled into the functional foregut during ingestion, where, over an unknown duration, the bacteria attach and form biofilms. *X*. *fastidiosa* is initially acquired onto low-flow regions of at least the precibarium during X wave testing behaviors; cibarial acquisition, while possible, was not considered in this work. Acquisition is dictated by rate-dependent bacterial attachment, while influenced by bacterial concentration and flowrates. During periods of non-probing behavior, bacteria multiply and twitch to form stochastic formations. During the next ingestion/egestion events, bacteria that have not reached tenacious configurations detach in most areas of the precibarium. This cyclical process of stochastic proliferation and patterned detachment continues until *X*. *fastidiosa* forms shag-carpet biofilms. All the while, bacteria spread to new locations. During egestion, high flowrates detach cells from the functional foregut and carry them into plant xylem vessels via fluid flow-driven detachment.

In conclusion, we simulated fluid dynamics during ingestion and egestion by extension of the cibarial diaphram, for both an accurate anatomical model of the functional foregut of *G*. *atropunctata* and xylem vessels of variable diameters. Our results support that flow-driven mechanisms are necessary and sufficient for vector acquisition and inoculation of *X*. *fastidiosa*; our work did not address possible involvement of saliva or variable amounts of uplift of the diaphragm that occurs. Moreover, the insect fluid dynamic simulations mirror known bacterial colonization patterns within the foregut, suggesting that fluid dynamics also shape retention within the functional foregut. Specifically, areas of predicted stagnation favored bacterial colonization and those predicted to experience high fluid shear forces had little to no bacterial colonization [6]. The 3D models in our insect simulations could be reapplied to simulate *G*. *atropunctata’*s feeding on different host plants. In addition, 3D models and simulations could be created for other vectors of *X*. *fastidiosa*, such as the meadow spittlebug, *Philaenus spumarius*, and *H*. *vitripennis*, which are implicated in other serious crop diseases caused by *X*. *fastidiosa* [43]. Future biological experiments that test this model will confirm the role that fluid flow rate play in the dynamics of bacterial adhesion to plant and insect surfaces during the transmission process.

## Methods

The results for our xylem and insect fluid dynamic simulations are reported as drag forces acting on individually surface-attached bacteria. Since it would be impractical to calculate these drag forces directly, a method was developed for estimating the mean drag force acting on bacteria that are hypothetically present in simulations. Drag forces were calculated using location-specific fluid velocities and shear rates. These calculations were informed by the work of De La Fuente et al. [29] and appear in the SI. Fluid dynamic simulation results and colonization patterns were plotted using MATLAB® (Mathworks Inc, Natick, MA).

### Xylem simulations

Fluid dynamics in xylem vessels were simulated in COMSOL Multiphysics^®^ (COMSOL, Inc., Stockholm, Sweden). 3D cylinders were used, with inlet and outlet specified based on the type of simulation. In the simulation, the boundary conditions were specified based on a water potential gradient of 0.03 MPa/m, which was previously reported for both well-irrigated and water-stressed grapevines. The water potential gradient was modeled as a pressure differential. In the *Ingestion* and *Egestion* simulations, flowrates were specified by dividing the calculated maximum volume of the insect cibarium according to our 3D model by the duration of filling and emptying of the cibarium during ingestion and swallowing according to EPG waveforms. Mesh refinement studies were completed for different vessel sizes and boundary conditions. More details on xylem simulations are in Section S3.1 in the S1 File.

### Insect simulations

Fluid dynamics in the precibarium were simulated in COMSOL Multiphysics® using our 3D model of the precibarium and cibarium [20]. The cibarial diaphragm was used as the source of pressure/tension. The cibarial diaphragm was used as the outlet (as a stand-in for the true mouth, for simplification) and the food canal opening at the stylet tips was used as the inlet during ingestion. The opening of the food canal was used as the outlet during egestion [20]. The flowrates calculated for *Ingestion* and *Egestion* xylem simulations were normalized based on the diaphragm surface area at maximum extension to calculate possible boundary velocities (indicated by blue coloring in S5B Fig in S1 File). These velocities were specified as perpendicular to the diaphragm to mimic the effects of diaphragm extension and relaxation. Simulation results and the 3D model meshes were exported and used in MATLAB® to calculate the drag forces acting on bacteria. The location of the bacteria was based on previous research displaying the locations of bacterial growth in the biofilm [6]. Mesh refinement studies were completed. The temperature of water in insect simulations was specified based on heat transfer simulations. The Newtonian slip length of water over the insect cuticle was accounted for, based on the slip length of a similar surface. More details on insect simulations are in Section S3. 2 in the S1 File.

## Supporting information

S1 File Supporting information for fluid dynamic simulations at the interface of the *G*. *atropunctata* functional foregut and grapevine xylem sap with implications for transmission of *Xylella fastidiosa*. (DOCX)
