# Supplementary material for: Fluid dynamic simulations at the interface of the blue-green sharpshooter functional foregut and grapevine xylem sap with implications for transmission of Xylella fastidiosa
Source: PLoS One. 2022 Mar 22;17(3):e0265762. doi: 10.1371/journal.pone.0265762 (PMC8939801; doi:10.1371/journal.pone.0265762)
Supplement: S1 File — (DOCX) [file pone.0265762.s001.docx]

**Supplemental Information for**

**Fluid dynamic simulations at the interface of the *G. atropunctata* functional foregut and grapevine xylem sap with implications for transmission of *Xylella fastidiosa***

Ian M. Marcus, Daniel White, Elaine A. Backus,

Sharon L. Walker, and M. Caroline Roper

Table of Contents

[Table of Contents 2](#_Toc88480296)

[S1 Results 5](#_Toc88480297)

[S1.1. Full Insect Simulations 5](#_Toc88480298)

[Figure S1. Full insect simulation results, including the precibarium, cibarium (with cibarial diaphragm fully extended), and true mouth. 6](#_Toc88480299)

[S1.2 Second View of Figure 3 Results 6](#_Toc88480300)

[Figure S2. Precibarial insect simulation results 7](#_Toc88480301)

[Table S1. Potentially useful quantities calculated from insect simulations. 7](#_Toc88480302)

[Table S2. Flow rates calculated for the *Undisturbed* xylem simulations. 8](#_Toc88480303)

[S2 Discussion 9](#_Toc88480304)

[S2.1 Timing of Bacterial Acquisition 9](#_Toc88480305)

[S2.2 Locations of Initial Bacterial Acquisition 11](#_Toc88480306)

[S3 Methods 12](#_Toc88480307)

[S3.1 Xylem Simulations 12](#_Toc88480308)

[S3.1a Illustrated Methods 12](#_Toc88480309)

[Figure S3. Arrangement for both undisturbed and disturbed xylem simulations. 12](#_Toc88480310)

[S3.1b Simulation Inputs 12](#_Toc88480311)

[S3.1c Calculation of Simulation Inputs 13](#_Toc88480312)

[Figure S4. 3D model of the blue-green sharpshooter cibarium used for estimating the cibarium volume 14](#_Toc88480313)

[S3.1d Sensor Locations 15](#_Toc88480314)

[S3.1e Mesh Refinement 16](#_Toc88480315)

[S3.1f Additional Assumptions 16](#_Toc88480316)

[S3.2 Insect Simulations 16](#_Toc88480317)

[S3.2a Illustrated Methods 16](#_Toc88480318)

[Figure S5. Contextualization of insect fluid dynamic simulations. 17](#_Toc88480319)

[Figure S6. Illustrated derivation of the 3D molds used for displaying colormaps of drag forces. 18](#_Toc88480320)

[S3.2b Simulation Inputs 19](#_Toc88480321)

[Table S3. Time required for minimum water temperatures in heat transfer simulations to approach within 0.1 °C of boundary temperatures. 23](#_Toc88480322)

[Figure S9. Temperature sensitivity analysis for insect (blue-green sharpshooter) fluid dynamic simulations. 26](#_Toc88480323)

[Figure S10. Mesh refinement study for the insect simulations. 28](#_Toc88480324)

[Figure S11. Location in the 3D model of the precibarium and cibarium with the highest calculated Reynolds number 30](#_Toc88480325)

[S3.3 Calculated Drag Forces 31](#_Toc88480326)

[S3.3a Summary 31](#_Toc88480327)

[S3.3b Illustrated and Detailed Methods 32](#_Toc88480328)

[Figure S12. Basic diagram of fluid flowing past a surface-attached bacterium 32](#_Toc88480329)

[Figure S14. Evaluation of the need for including pressure drag in total drag force calculations 34](#_Toc88480330)

[Figure S16. Correlation between flow properties (*ν^2^* and *dν/dy*) and drag force (*F*) in our microfluidic chamber simulations. 36](#_Toc88480331)

[Table S4. Shear rates (s^-1^) in microfluidic chamber simulations. 40](#_Toc88480332)

[Figure S17. Cross-section of the precibarium used for water hammer calculation. 41](#_Toc88480333)

[References 43](#_Toc88480334)

# S1 Results

## **S1.1. Full Insect Simulations**

For all figures mentioned herein, a number not preceded by S refers to a figure in the main article, and a number preceded by S refers to a figure in this Supplemental Information.

While Figure 3 displays insect simulation results that pertain to the precibarium, Figure S1 displays the full insect simulation results, with both the precibarium and cibarium. Predicted mean drag forces are low almost everywhere except in the precibarium.


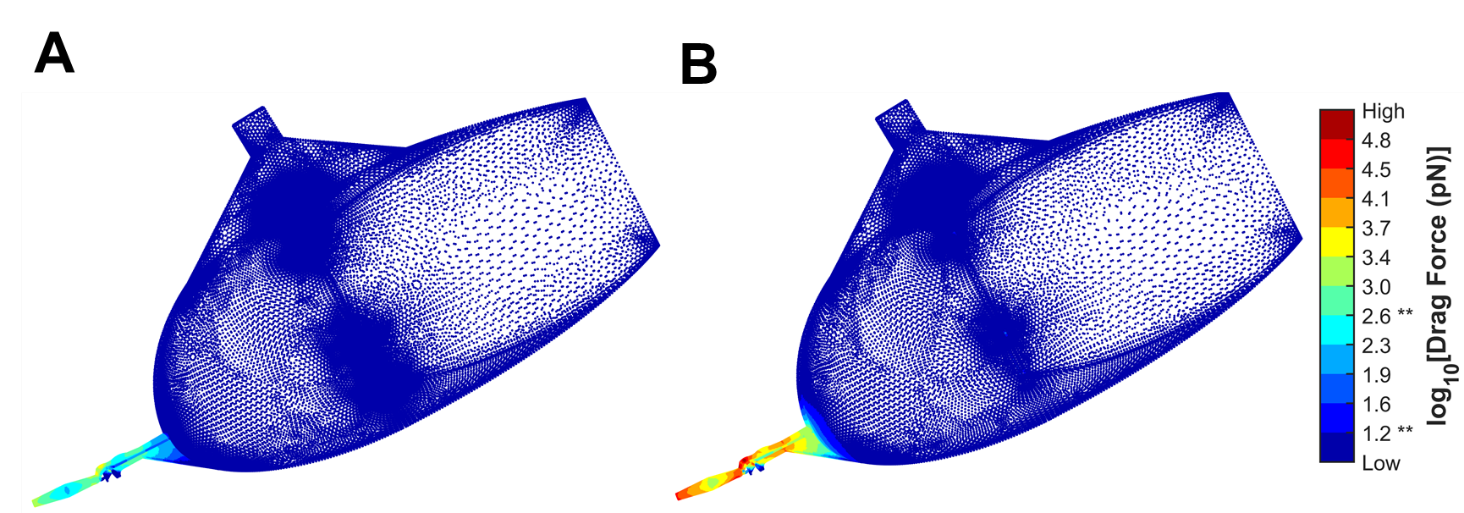


Figure S1. Full insect simulation results, including the precibarium, cibarium (with cibarial diaphragm fully extended), and true mouth.

The results for the precibarium are examined closely in Figure 3. These colormaps are of simulated mean drag forces acting on individually surface-attached bacteria in the blue-green sharpshooter during feeding. During ingestion (A), drag forces are generally lower than during egestion (B). The ** symbols on the colormap are references to key drag forces in the results of Fuente et al. [1]. 10^1.2^ pN (15.85 pN) represents the minimum drag force that caused meaningful detachment of individually surface attached *Xylella fastidiosa*. When the drag forces in the referenced work were incrementally increased, representative results showed that over 70% of *X. fastidiosa* detached by the time the drag forces reached 10^2.6^ pN (443.8 pN) [1].

## S1.2 Second View of Figure 3 Results

Figure 3 displays insect simulation results that pertain to the precibarium. Figure S2 displays precibarium results from another viewpoint that is useful for analyzing results.


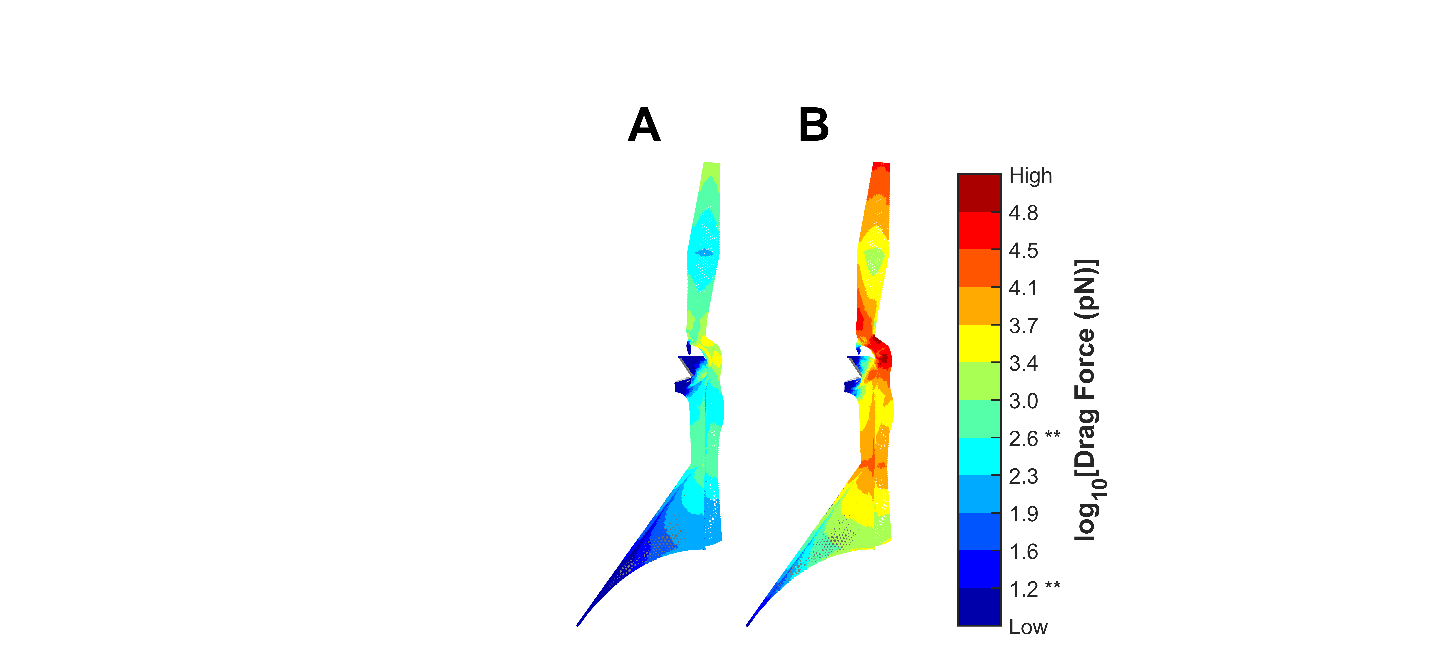


**Figure S2**. Precibarial insect simulation results, displayed in a different arrangement (side view) than in Figure 3 of the main text.

The hypopharyngeal and epipharyngeal halves of the precibarium are apposed, with only the left half of the precibarium visible. During ingestion (A), simulated drag forces are generally lower than during egestion (B).

S1.3 Generally Useful Simulation Results

Table S1. Potentially useful quantities calculated from insect simulations.

| Cibarium volume^[[1]](#footnote-1)^ (nL) with diaphragm fully extended | 28.6 |
| --- | --- |
| Precibarium volume (nL) | 0.0558 |
| Surface area of precibarium (µm^2^) | 14241 |
| Surface area of precibarium + cibarium + true mouth (µm^2^) | 625070 |
| Ingestion mean flow rate (m^3^/s) | 3.37*10^-11^ |
| Egestion mean flow rate (m^3^/s) | 4.80*10^-10^ |

Table S2. Mean flow rates calculated for the *Undisturbed* xylem simulations.

| Vessel Diameter (µm) | Flowrate (nL/s) |
| --- | --- |
| 20 | 0.1 |
| 30 | 0.6 |
| 40 | 1.9 |
| 50 | 4.6 |
| 60 | 9.7 |
| 70 | 17.9 |
| 80 | 30.7 |
| 90 | 49.4 |
| 100 | 76.0 |
| 110 | 111.4 |
| 120 | 157.0 |
| 130 | 236.6 |
| 140 | 321.4 |
| 150 | 384.8 |
| 160 | 502.9 |
| 170 | 639.1 |
| 180 | 804.0 |
| 190 | 998.7 |
| 200 | 1228.8 |

# S2 Discussion

## S2.1 Timing of Bacterial Acquisition

Soon after the start of stylet probing, a blue-green sharpshooter begins searching for a mature xylem vessel for ingestion. In electropenetrographic (EPG) recordings, this is called Pathway phase. Small amounts of fluid uptake and egestion for tasting/testing xylem vessels occur during pathway. However, most xylem vessels visited in that phase are small, immature vessels that are rejected by the sharpshooter [2, 3]. When a wider, more acceptable, mature xylem vessel is found, the insect performs more extensive testing for acceptance. Termed the X wave, this testing consists of two, temporally alternating parts (XNC and XC2) [4]. XNC represents rapidly repeated, sequential behaviors: 1) salivation (both watery and gelling saliva; gelling saliva reinforces the salivary sheath connection to the xylem vessel, watery saliva is taken back up), 2) uptake of mixed saliva-xylem fluid into just the precibarium (micro-ingestion), 3) swishing the fluid around, then 4) the fluid being egested out, either slowly (“dribbling”) or very quickly/strongly (“spitting”). XC2 represents trial ingestion, with 3 to 300 cibarial pulls/fills and swallows (each represented by a single plateau in the series of plateaus during XC2) within 5 min, to test the mechanical, salivary sheath connection to the xylem vessel.

Consequently, to simplify our model, we assumed that bacteria are most likely acquired into the precibarium during contact with wider, mature xylem cells represented by the X wave, for multiple reasons. The highest drag forces likely occur during the in-and-out fluid motions occurring during XNC, alternating with XC2. Thus, XNC/XC2 likely creates harsher flow environments for xylem biofilms than later sustained ingestion (waveform C2, described further, below) (Figure. 2). This is because C2 does not include egestion, but instead causes more of an always-inward, start-stop motion as fluid is taken up, then swallowed. This means the maximum xylem drag forces are predicted to be 14 times higher for the strongest “spitting”-type of egestion during XNC than during trial ingestion in XC2 (or sustained ingestion, C2, later) in the vessels, where ingestion is also relevant for bacteria detachment from xylem vessel walls. Harsher flow conditions cause more xylem-dwelling bacteria to detach from the xylem element walls and enter the functional foregut as planktonic bacteria and floating bacterial aggregates. Higher floating bacterial concentrations cause more attachment in the precibarium, since bacterial deposition rates can be approximated as being proportional to floating bacterial concentration [5].

X wave tasting/testing (alternating XNC/XC2 events) always precedes sustained ingestion (C2) in a selected xylem vessel [6]. After X wave testing/tasting is completed and a mature xylem vessel is accepted, continuous fluid-uptake and swallowing (sustained ingestion, C2) begins; these continuous cibarial pumps can continue for 2 – 5 h. Any bacteria prone to detachment may already be detached during XC2 before the thousands of C2 swallows commence. Conversely, multiple egestion spurts (“spits”) typically occur during one XNC event, between XC2 events. This means planktonic bacteria concentrations can build up between egestion spurts, and lead to higher deposition rates in the functional foregut during XC2.

Notwithstanding the above, it is likely that bacterial acquisition in the precibarium occurs mainly during C2 ingestion instead of XC2 ingestion. As discussed in the results, water hammer shockwaves could travel from the precibarial valve, through the stylet food canal, and into the xylem to detach bacteria. Water hammer likely occurs during both XC2 and C2 ingestion. Yet, C2 sustained ingestion lasts for many hours, while each XC2 trial ingestion~~s~~ event lasts for no more than 5 min [6]. Longer durations may allow for more bacterial acquisition in the proximal regions of the precibarium during C2 ingestion, due to the rate-based nature of bacterial deposition [5]. Therefore, the timing of when the bulk of bacteria acquisition occurs is a tradeoff between fluid dynamics, insect anatomy, xylem anatomy, and insect stylet probing behaviors.

## S2.2 Locations of Initial Bacterial Acquisition

Based on current results (Figure 3 and Figure 4), initial bacterial acquisition in the precibarium is mainly expected to occur near the precibarial pit/ring, the valve/flap shadow, and the cibarial epipharyngeal trough. Acquisition is expected in these low-flow precibarial regions during ingestion and egestion because shear forces required to prevent bacterial deposition are generally lower than those required to detach the same bacteria [7]. According to EPG recording, there sometimes are 0.5 to 5 second pauses between the cibarial fills/swallows during XC2 that could greatly increase the surface area in the precibarium available for attachment [6]. However, these pauses are not expected to greatly alter the sites of initial acquisition. In the case in which bacteria attach under the inferred quiescent conditions of such XC2 pauses, the majority are likely swept away by the ensuing ingestion flowrates, because bacteria typically exhibit bond-strengthening with a surface on a time-scale of minutes after initial adhesion [5]. If anything, acquisition during XC2 pauses is mainly expected to expand the region of acquisition in the precibarial trough, which has a relatively low gradient of predicted drag forces (Figure 3). Therefore, most initial acquisition is inferred to occur in low-flow regions of the precibarium.

# S3 Methods

## S3.1 Xylem Simulations

### S3.1a Illustrated Methods

Fluid dynamics in grapevine xylem vessels were simulated in COMSOL Multiphysics^®^ (COMSOL, Inc., Stockholm, Sweden), as illustrated in Figure S3.


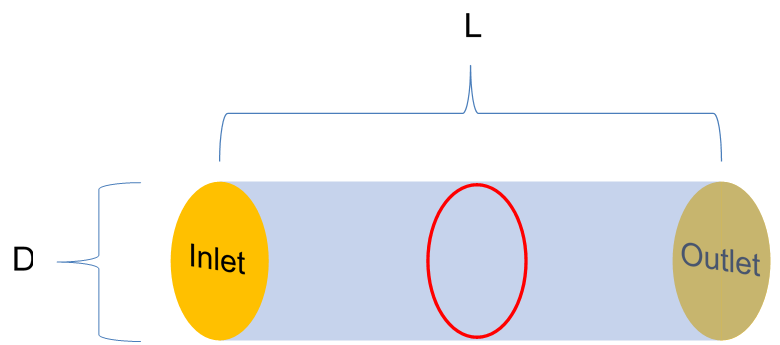


### Figure S3. Arrangement for both undisturbed and disturbed xylem simulations.

3D cylinders were used, with inlet and outlet specified based on the type of simulation. The length (L) of cylinders was 100 µm. The diameter (D) of cylinders was increased stepwise from 20 µm to 200 µm by increments of 10 µm using a parametric sweep for each simulation. At the midpoints of the cylinders, edge probes (red) were used to calculate velocities and shear rates near the walls of the cylinder, which were used to calculate the drag forces acting on individually surface-attached bacteria. The edge probes were in the shape of circles with radii equal to that of the cylinder, minus 0.2887 µm.

###

### S3.1b Simulation Inputs

In the *Undisturbed* simulation, the boundary conditions were specified based on a water potential gradient of 0.03 MPa/m, which was previously reported for both well-irrigated and water-stressed grapevines [8]. The water potential gradient was then used to calculate the pressure drop across the pipe ((0.03 MPa/m)*(100e-6 m) = (3e-6 MPa)). The inlet and outlet were specified to have fully developed flow with average pressures corresponding to the calculated pressure drop.

In the *Ingestion* and *Egestion* simulations, flow rates were specified by dividing the calculated volume of the insect cibarium by the duration of filling and emptying of the cibarium during EPG waveform C2. Next, these flow rates were divided by two to account for an assumed equal partitioning of flow in xylem vessels upstream and downstream of penetrating stylets. This assumption can be used because the forces caused by insect feeding dominate in the xylem. The water potential gradient due to evapotranspiration would affect the partitioning rather than the magnitude of tension in the xylem. Figure 2 demonstrates that drag forces in xylem are much higher during feeding than in undisturbed xylem. Thus, it is reasonable to assume that the tension gradient inside xylem would only affect the partitioning in minor ways. Changing the partitioning would only strengthen the conclusions of the manuscript, because if all the flow would go in one direction inside the xylem, it would increase the drag forces. Thus, it would still demonstrate that insect feeding detaches bacteria inside the xylem. The xylem vessel (cylinder) inlets were specified to have fully developed flow with flow rates of (1.685 e-11 m^3^/s) and (2.4e-10 m^3^/s) for *Ingestion* and *Egestion* simulations, respectively.

### S3.1c Calculation of Simulation Inputs

In the *Ingestion* and *Egestion* simulations, flowrates were specified by dividing the calculated volume of the insect cibarium by the durations of filling and emptying of the cibarium during C2, and then dividing by two. The volume was calculated from our 3D model of the cibarium (with the cibarial diaphragm at maximum expansion based on diaphragm measurements) [9], and thus represents the maximum volume possible. The durations were estimated based on published EPG recordings [6].

Our 3D model of the cibarium accounted for five dimensions aimed to mimic the cibarium of blue-green sharpshooters [9]. Our calculated volume ignored the volume added to the cibarium by the precibarium, longitudinal groove, and true mouth, as shown in Figure S4. Using COMSOL Multiphysics^®^, a volume of 2.86e-11 m^3^, or 28.6 nanoliters was calculated. This calculated volume is comparable to the 8.7e-11 m^3^ reported elsewhere based on a different volume estimation method [10].


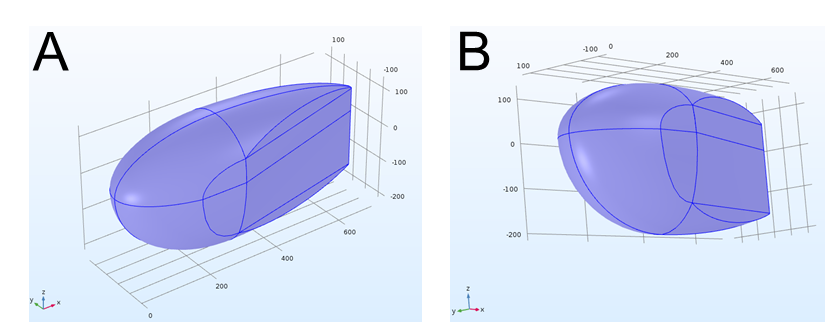


**Figure S4**. 3D model of the blue-green sharpshooter cibarium used for estimating the cibarium volume. The precibarium, longitudinal groove, and true mouth geometries were not included in this calculation. The model is shown from two different angles in COMSOL Multiphysics^®^. It has a calculated volume of 28.6 nanoliters.

The durations of ingestion and egestion required to fill and empty the cibarium were estimated from published EPG recordings of blue-green sharpshooters feeding on grapevine [6]. The flowrates of ingestion and egestion during components of the X wave, XNC (egestion) and XC2 (trial ingestion), were assumed to be the same as the flowrates of ingestion and swallowing during C2 (sustained ingestion). Estimations were based on C2, because the regularity of the C2 waveform allows for easier estimation of the time required to fill and empty the cibarium.

The repetition rate for C2 plateaus was reported as 1.1 Hz by Cervantes and Backus (Figure 4 in reference 6) under the high biopotential (emf)-sensitive conditions of 10^9^ Ohms input resistor, applied AC signal to the plant, and silver glue wire adhesive on the insect [6]. However, the previous recording did not include a peak representing closure of the precibarial valve. In contrast, their Figure 9 illustrating C2 EPG recordings included such a peak because they were recorded under high-resistance component conditions of 10^7^ and 10^8^ Ohms, applied AC, and silver paint (Figure 9 in reference 6). Using the bulk of the recorded plateau as ingestion, and the R-component peak as representative of swallowing/simultaneous precibarial valve closure, we estimated the ratio of the duration of C2 ingestion and swallowing as 14.24:1.

Therefore, the estimated durations of C2 cibarium filling and emptying are 0.849 s and 0.0597 s, respectively. These durations, together with the cibarial volume, yield estimated ingestion and egestion flow rates of 3.37e-11 m^3^/s and 4.80e-10 m^3^/s, respectively. These flowrates were divided by two for xylem vessel *Ingestion* and *Egestion* simulations, because it was assumed that flow is equally partitioned upstream and downstream of penetrating stylets. We justified this assumption because calculated flowrates were high enough that the insect could essentially overcome natural xylem flow from evapotranspiration, thus equalizing flow in both directions.

~~but~~ We do not expect that our calculated drag forces are overpredicted. We have made order of magnitude predictions of drag forces, so cibarial filling assumptions between 10% and 100% are not expected to change the order of magnitude of flowrate. The flowrate that has the largest effect on detachment during ingestion is likely the peak flowrate. The peak flowrate is higher than the ~~average~~ mean flowrate because the cibarial diaphragm movement during ingestion would likely be slower at the beginning and end of cibarial diaphragm uplift. Because our simulations are based on the predicted ~~average~~ mean flowrate, they likely underpredict the flowrates that impact detachment of bacterial cells from xylem walls the most. In addition, our simulations use the average duration of C2 episodes, as measured by electropenetrography (EPG). In reality, there is likely variability in flowrates between C2 episodes, and the influential peak flowrates are underestimated when we use average durations. Finally, our egestion flowrates are especially underestimated because they are based on the duration of swallowing. During swallowing, cibarial diaphragm recoil works against the esophageal valve. During egestion, cibarial diaphragm recoil works in the same direction as xylem sap tension, presumably leading to higher flowrates during egestion than during swallowing.

Our calculated values for cibarial volume and ingestion flowrate are relatively small compared to values published by Ranieri, et al. [10], who used µ-CT when computing cibarial volume. Here is a quick comparison between our values and theirs:

1.
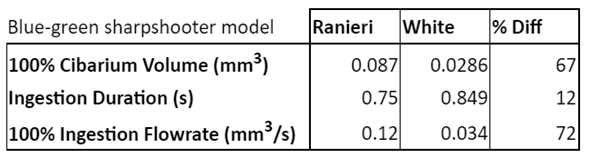


**Marcus**

Our ingestion flowrate corresponds with 28% filling of the cibarium model presented by Ranieri, et al. [10], which is toward the lower range of their modeled flowrates. Thus, if anything, our predicted flowrates are conservative.

### S3.1d Sensor Locations

As illustrated in Figure S4, velocities and shear rates in the fluid dynamic simulations of xylem vessels were probed 0.2887 µm from the cylinder walls. Concentric ring edge probes were used in simulations to avoid inconsistencies associated with point sensors, which yield slightly variable measurements depending on their angular position. This variability may be due to the limitations of finite element calculations in the fluid dynamic simulations, which use a mesh-based method. The concentric ring edge probes were positioned at the midpoint of the 100 µm long cylinders to avoid entrance and exit effects.

### S3.1e Mesh Refinement

Xylem simulations used “Fine” physics-controlled meshes in COMSOL Multiphysics^®^. The accuracy of the “Fine” mesh setting was evaluated by comparing results between this mesh setting and the next-best mesh setting, “Normal.” Results were compared at a variety of cylinder diameters, a variety of calculated drag forces, and both types of boundary condition sets by running the *Undisturbed* and *Egestion* simulations with “Normal” physics-controlled meshes. Results with “Normal” meshes were within 8% of results with “Fine” meshes. On average, there was a 1% difference between “Fine” and “Normal” results.

### S3.1f Additional Assumptions

1. The viscosity of xylem sap is the same as that of water [11]. Xylem sap is an incompressible [12] Newtonian [13] fluid that can be modeled by the Navier-Stokes equations at the size scale of interest [14].
2. Xylem flow is laminar and steady state, and there is no slip at xylem vessel walls.
   1. Laminar flow was verified based on the *Egestion* simulation of the 20 µm xylem vessel. The Reynolds number was calculated to be 15.

## S3.2 Insect Simulations

### S3.2a Illustrated Methods

Fluid dynamics in the blue-green sharpshooter precibarium and cibarium during feeding were simulated in COMSOL Multiphysics^®^ (COMSOL, Inc., Stockholm, Sweden), as illustrated in Figure S5.


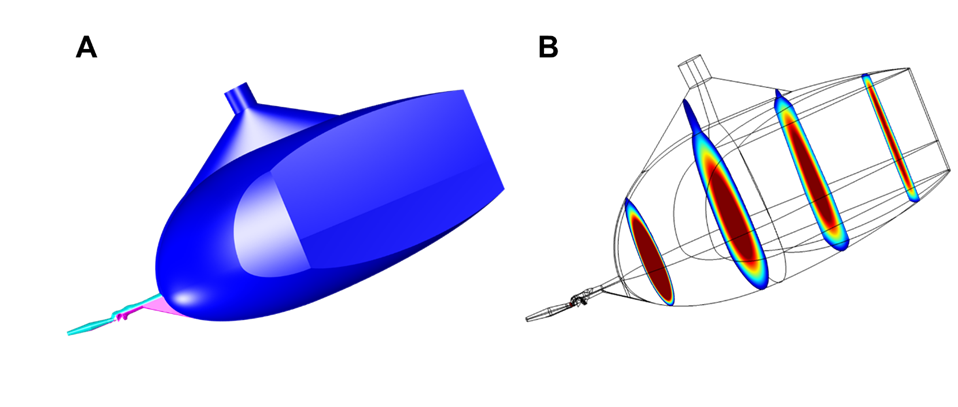


### Figure S5. Contextualization of insect fluid dynamic simulations.

A) The 3D model of the blue-green sharpshooter precibarium and cibarium, which was described in another study using different coloring to label the model [9]. The precibarial epipharynx (magenta) and precibarial hypopharynx (cyan) are emphasized here as the portions of the 3D model used in Figure 3. The blue portion of the 3D model includes the cibarium and true mouth, in which fluid dynamics were also simulated, but the results for this region are only included in the supporting materials (herein). B) Fluid dynamics were simulated in the whole 3D model, including the precibarium, cibarium, and true mouth. Red regions in the slice plot indicate areas with high fluid velocities and blue regions indicate regions with low (boundary) velocities. Full fluid dynamic simulation results are described in more detail above.

The simulation results presented in the main text are focused on the precibarium of the insect, as illustrated in Figure S6.


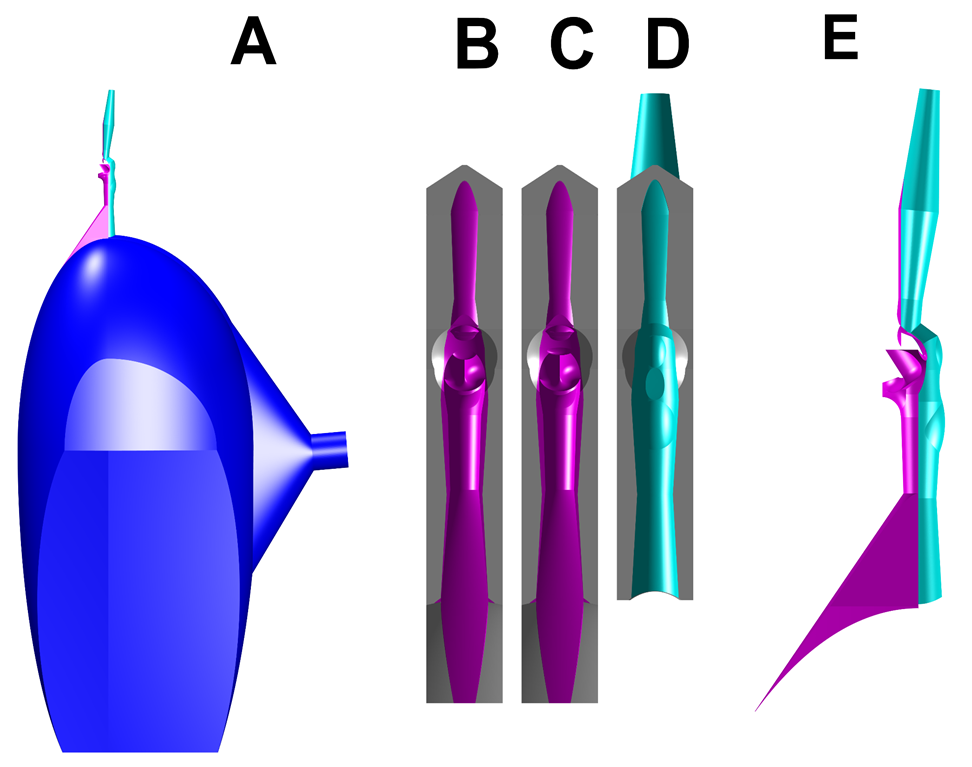


### Figure S6. Illustrated derivation of the 3D molds used for displaying colormaps of drag forces.

A) The 3D model of the blue-green sharpshooter precibarium and cibarium shown in Fig S4A, rotated for comparison with subsequent subfigures. B) The precibarial epipharynx (magenta) from subfigure A plotted within a gray representation of a portion of the epipharynx. The precibarial valve is in the same position as in the previously reported 3D model [9]. C) A very similar 3D mold to that presented in subfigure B, except that a portion of the precibarial valve is sticking out into the lumen of the precibarium. This represents the inferred position of the precibarial valve during egestion, due to fluid drag. The precibarial valve of aster leafhoppers has been described as hollow and mainly composed of cuticle [15], so we infer that the blue-green sharpshooter precibarial valve is flexible and deformed during outflow. The deformed precibarial valve was included in the 3D model used for the egestion simulation. The 3D models used for ingestion and egestion simulations were different only in this respect. D) The precibarial hypopharynx (cyan) from subfigure A plotted within a gray representation of a portion of the hypopharynx. E) Half of the precibarial epipharynx and hypopharynx from subfigure A. This portion of the 3D model provides an alternative way of presenting colormaps of drag forces.

The cibarial diaphragm was used as the outlet and inlet for the ingestion and egestion simulations, respectively, as illustrated in Figure S7.


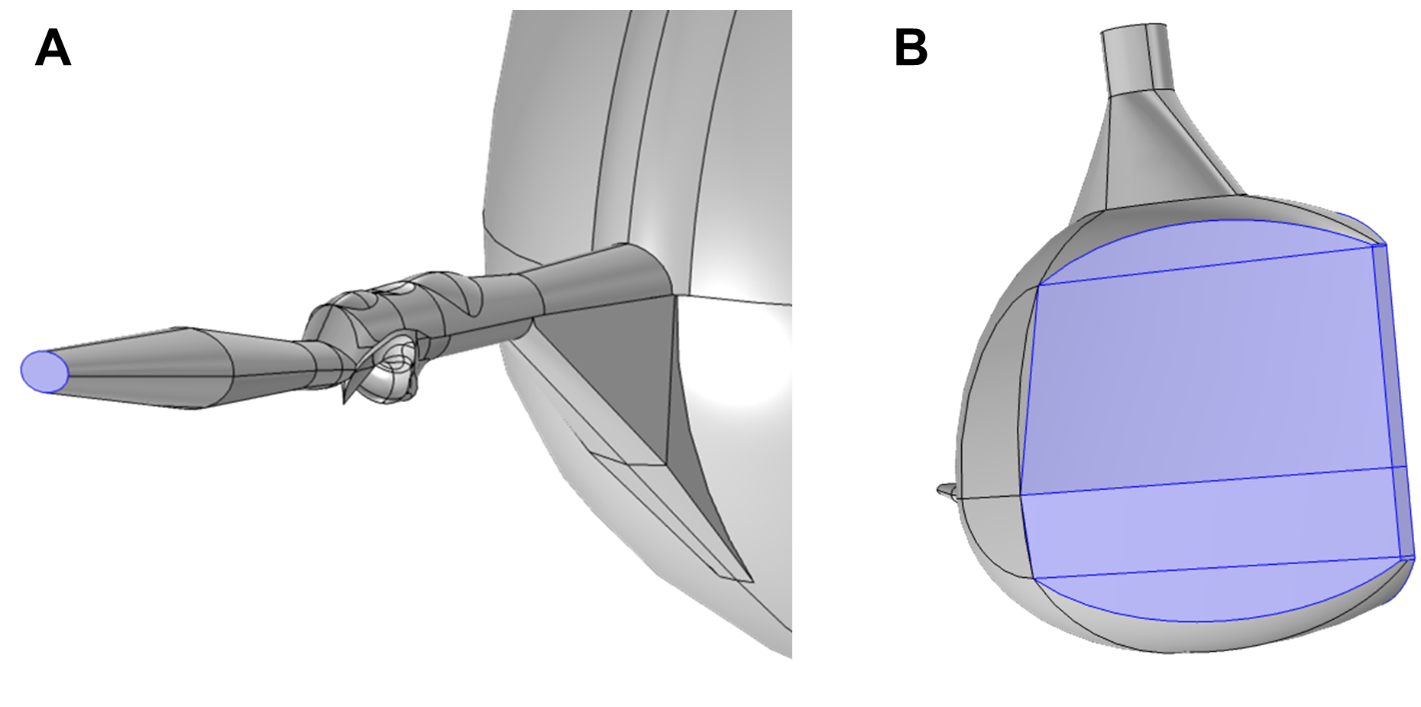


Figure S7. The inlet (A) and outlet (B) used for the ingestion simulation, indicated in blue.

The blue regions identify the entrance to the HEF (A) and a symmetrical section of the cibarial diaphragm (B). The inlet and outlet were reversed (swapped) for the egestion simulation. HEF: hypopharyngeal extension that inserts into the stylet food canal.

### S3.2b Simulation Inputs

In the ingestion and egestion simulations, fluid velocities were specified for the section of the cibarial diaphragm indicated in Figure S7. This indicated section served as the outlet for the ingestion simulation and the inlet for the egestion simulation, simulating the effects of the cibarial diaphragm being fully extended and released by the cibarial dilator muscles. Specified velocities were 0.14 mm/s and 2.0 mm/s for the ingestion and egestion simulations, respectively.

Calculations and Validation of Simulation Inputs

Specified velocities at the cibarial diaphragm are derived from the ingestion and egestion flowrates calculated above (section S3.1c). These flowrates were divided by the surface area of the indicated section of the cibarial diaphragm (Figure S7B) to calculate boundary velocities:

$$\hat{Q}_{boundary\_ingestion}=\frac{3.37*{10}^{-11}\frac{m^{3}}{s}}{2.4*{10}^{-7}m^{2}}=1.4*{10}^{-4}\frac{m}{s}$$

$$\hat{Q}_{boundary\_egestion}=\frac{4.80*{10}^{-10}\frac{m^{3}}{s}}{2.4*{10}^{-7}m^{2}}=2.0*{10}^{-3}\frac{m}{s}$$

Boundary velocities were specified as perpendicular to the cibarial diaphragm to mimic the effects of diaphragm extension and relaxation.

Temperature: Heat Transfer Simulation

The temperature of xylem sap in insect fluid dynamic simulations was carefully chosen to be 20 °C because the viscosity of sap is affected by its temperature, which is affected by the insect temperature. The chosen sap temperature is a Fermi estimate based on heat transfer simulations and an analysis that accounts for sharpshooter feeding behavior. The Fermi estimate is explained below, followed by a sensitivity analysis of insect fluid dynamic simulations with respect to sap temperature.

Heat transfer simulations predict that the xylem sap in the precibarium and cibarium is at the same temperature as the insect. Before sap enters the precibarium, it travels through the stylet food canal and is likely warmed by the partially ensheathing labium of the insect, which we infer has a similar temperature to the insect body. In blue-green sharpshooters, the stylet food canal has a mean area of 28 µm^2^ and the labium is around 650 µm long [10, 16]. Since the ingestion flowrate is estimated to be 3.37*10^-11^ m^3^/s, the average velocity in the stylet food canal while the xylem sap is warming is 1.2 m/s. The maximum velocity is twice the average velocity, or 2.4 m/s, assuming Hagen Poiseuille flow. This means the fastest moving xylem sap spends 0.27 milliseconds moving through the stylet bundle.

Our time-dependent heat transfer simulations predict that xylem sap reaches the temperature of the containing walls’ boundary layer by the time it enters the precibarium. Time-dependent heat transfer simulations were conducted for a cylinder of stationary water with a constant temperature boundary condition, as shown in Figure S8.


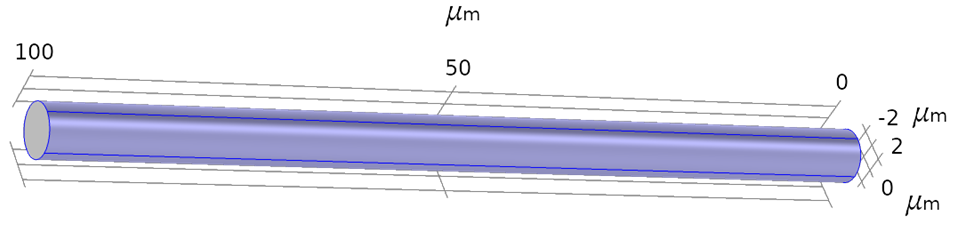


**Figure S8.** Illustration of the heat transfer simulation in COMSOL Multiphysics^®^.

Heat transfer was simulated between a solid cylinder of water and its constant temperature boundary, indicated in blue. The ends of the cylinder (gray) were specified as thermal insulation. The cylinder had a radius of 3 µm and a length of 100 µm. While the *Heat Transfer in Solids* interface was used, the properties of liquid water were used in the simulation.

The constant temperature boundary condition was specified as 14 °C higher than the initial temperature of the water based on the maximum expected temperature difference between the insect and xylem sap. This maximum expected temperature difference was estimated based on the size of the insect, its temperature difference from ambient air, and the temperature difference between air and xylem sap.

The mass of blue-green sharpshooters was estimated based on the mass of glassy-winged sharpshooters. Since glassy-winged sharpshooters that fed on grapevines had dry weights of 10 – 15 mg [17], and because blue-green sharpshooters are around half the size of glassy-winged sharpshooters, the mass of blue-green sharpshooters is estimated as 10^-5^ kg.

The maximum expected difference between ambient temperature and blue-green sharpshooter body temperature was estimated using measurements made on another insect that is also in the hemipteran infraorder Cicadamorpha, the citrus cicada, *Diceroprocta apache* (Davis)[18]. The body mass of *D. apache* was measured as 7*10^-5^ kg, so the maximum body-air temperature difference for this insect (12 °C) is expected to be greater than that for blue-green sharpshooters, since larger insects generally have larger maximum body-air temperature differences. Therefore, 12 °C is a conservative estimate for the maximum temperature difference between a blue-green sharpshooter and ambient air.

The temperature difference between ambient air and xylem sap was estimated for laboratory conditions where plants may be cooler than air due to transpiration and a lack of solar radiation. A previous study cited how elevated atmospheric concentrations of carbon dioxide causes partial stomatal closure, leading to reduced transpiration from plant leaves [19]. Associated increases in canopy temperature by 0.3 – 1.7 °C have been observed [19]. This supports our choice of a 2 °C temperature difference between xylem sap and air. Our estimate of a plant temperature lower than air is conservative since plant tissue temperatures have been reported as higher than air temperatures [20, 21].

By adding 12 °C and 2 °C together, we estimate the maximum temperature difference between insect and xylem sap to be 14 °C. This was the temperature difference used in the heat transfer simulations. Results are listed for two different sets of boundary conditions in Table S3.

### Table S3. Time required for minimum water temperatures in heat transfer simulations to approach within 0.1 °C of boundary temperatures.

| **T_boundary_initial_ (°C)** | **T_water_initial_ (°C)** | **Time (milliseconds)** |
| --- | --- | --- |
| 37 | 23 | 0.089 |
| 21 | 7 | 0.091 |

Less than 0.27 milliseconds are required for the temperature of the xylem sap in the stylet food canal to reach that of the boundary layer coating stylet food canal walls. Xylem sap draws heat out of the surrounding insect tissue. Based on the following analysis, we infer that surrounding insect tissue, and maybe the whole insect body, falls to the temperature of xylem sap inside the plant (undisturbed xylem sap).

When feeding on grapevine, glassy-winged sharpshooters consume around 20 – 30 times their dry body weight per hour [17, 22]. We presume the same is true for blue-green sharpshooters. Based on this large fluid intake and the large heat capacity of water, we infer that heat flows from insects to the xylem sap they consume until the portions of the insect in close proximity with the sap reach the same temperature as xylem sap inside the plant. This inference is supported by the increase in glassy-winged sharpshooter optimal survival temperature by 20 °C when fed on a host plant instead of no host plant [23].

In summary, we infer that xylem sap in the precibarium and cibarium of the blue-green sharpshooters during feeding is usually the temperature of xylem sap in the plant. Since the insect fluid dynamic simulations were created using experimental EPG data, a plant temperature was chosen to approximate conditions during the EPG study. The study was likely conducted at room temperature, with the plants likely being slightly cooler than room temperature due to evapotranspiration and a lack of solar radiation. Unless otherwise specified, fluid dynamics simulations in this work used a water temperature of 20 °C (for both insect simulations and xylem simulations).

The temperature of xylem sap flowing through the precibarium and cibarium likely varies due to differences in ambient temperature, how long an insect has been feeding, and more. In Figure S9, we compare insect fluid dynamic simulations at different temperatures to test the sensitivity of our simulation results. Temperature has little effect on simulation results.


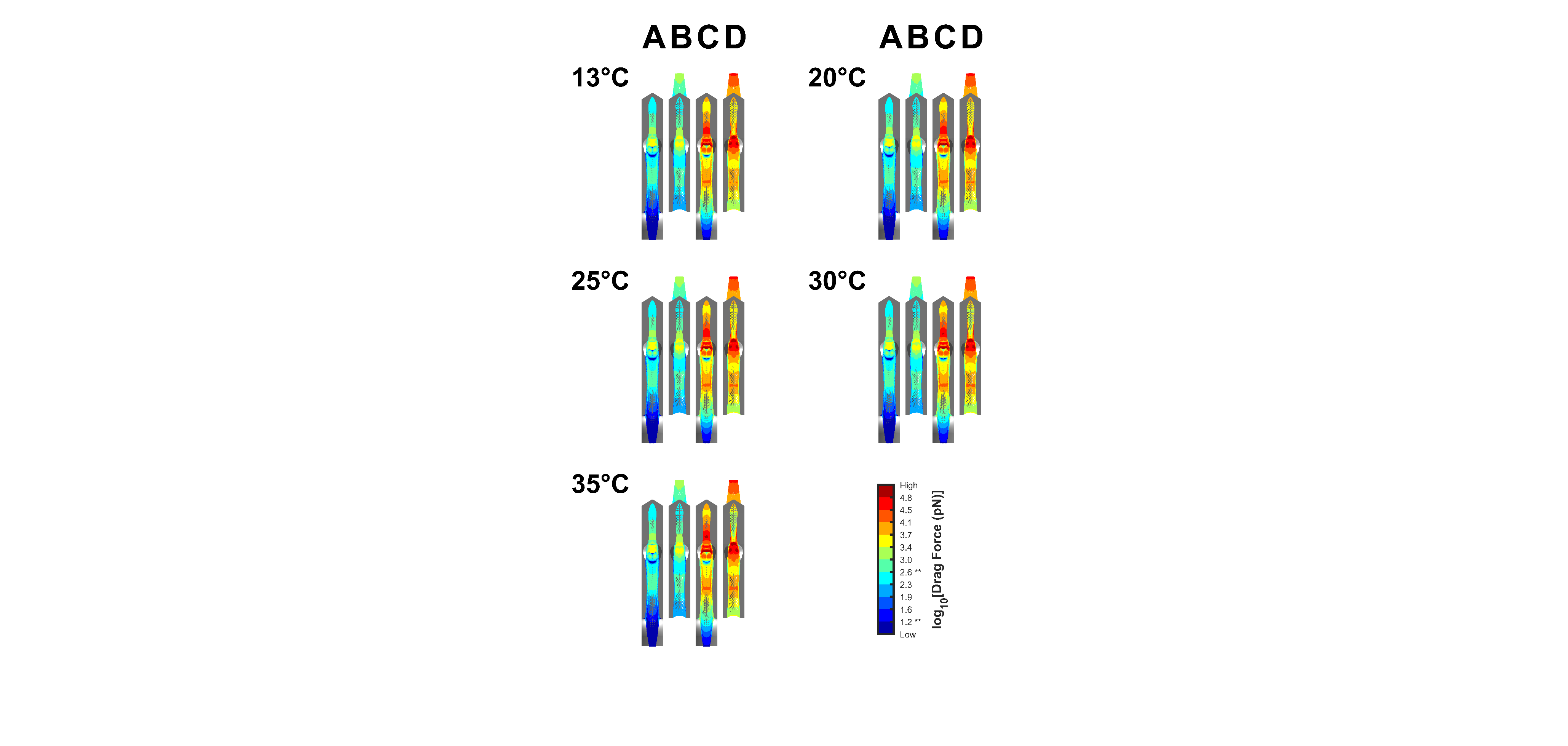


### Figure S9. Temperature sensitivity analysis for insect (blue-green sharpshooter) fluid dynamic simulations.

The simulation was run using xylem sap (water) at different temperatures. The 20 °C simulation results are presented in detail in Figure 3 of the main text. 13 °C – 35 °C is the range where glassy-winged sharpshooters produced excreta (fed on a host plant) in a previous study [24]. According to a previously published simulation, the temperature range of around 20 °C – 30 °C is optimal for glassy-winged sharpshooter survival on a host plant [23]. The 20 °C and 30 °C simulation results are very similar.

Slip Length

The no-slip boundary condition has traditionally been specified for most macroscopic flows [25]. In our insect simulations, this idealistic boundary condition could not be used. This is because we assumed that intima lining of the precibarium and cibarium had a waxy layer, similar to outer body cuticle; thus, the intima would be highly hydrophobic [26, 27]. Also, there was a need for precise simulation of fluid flow at the size scale of individual surface-attached bacteria. A Navier slip length of 28 nm was specified as a wall boundary condition in COMSOL Multiphysics^®^.

Thus, the functional foregut cuticle was expected to be smooth [28] and hydrophobic [26], with a contact angle around 105° [26, 29, 30]. The boundary slip length of water near a smooth surface with a contact angle of 98° was measured as 28 nm using colloidal probe of atomic force microscope [31, 32].

Probe Locations

Velocities and shear rates in insect fluid dynamic simulations were probed 0.2887 µm from the walls. This was accomplished by using both COMSOL Multiphysics^®^ and MATLAB. COMSOL was used to create meshes that mimicked those used for fluid dynamics simulations. These representative meshes were exported from COMSOL as .stl files and interpreted in MATLAB as triangulations. The incenters of each triangle were identified and points that were 0.2887 µm away, on triangle face normal vectors, were identified. These points were used as evaluation points in COMSOL Multiphysics^®^ to export velocities, shear rates, and calculated drag forces.

Mesh Refinement

Insect simulations used “Finer” physics-controlled meshes in COMSOL Multiphysics^®^. The “Finer” mesh setting was evaluated by comparing (Figure S10) results between this mesh setting and the next-best mesh setting “Fine” (except that an alteration was made to the “Fine” setting: the “Resolution of Narrow Regions” was changed from 0.7 to 0.72).


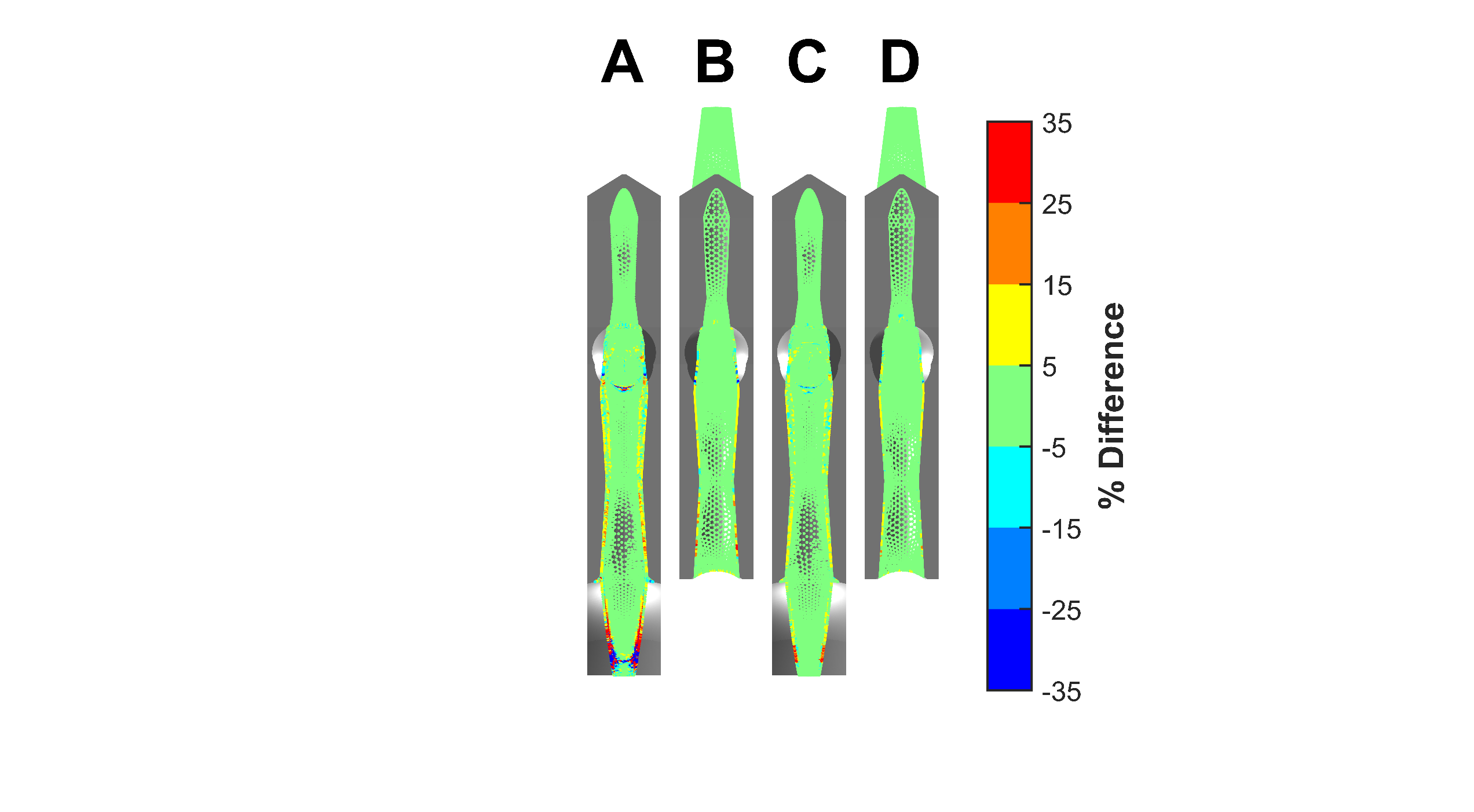


### Figure S10. Mesh refinement study for the insect simulations.

The results in Figure 3 are compared with results from lower resolution simulations. A) Ingestion, epipharynx, B) Ingestion, hypopharynx, C) Egestion, epipharynx, D) Egestion, hypopharynx. The simulations in Figure 3 used “Finer” physics-controlled meshes in COMSOL Multiphysics^®^. The lower resolution simulations used “Fine” physics-controlled meshes, with the “Resolution of Narrow Regions” changed from 0.7 to 0.72. Percent difference is displayed between the log_10_(Drag Force [pN]) of the “Finer” and “Fine” simulations. Most of the results for the lower resolution simulations are green because they are within 5% of Figure 3 results. Percent differences outside this range are often at stagnation zones (a small change in small drag force could be a big % difference) and sharp turns in the geometry (this may be due to the method of comparing meshes: the “Fine” simulations used the same probe locations as the “Finer” simulations).

Additional Assumptions

1. The viscosity of xylem sap is the same as that of water [11]. Xylem sap is an incompressible [12] Newtonian [13] fluid that can be modeled by the Navier-Stokes equations at the size scale of interest [14].
2. Flow is quasi-steady state.
   1. This assumption is not ideal due to the short duration of ingestion and egestion during sharpshooter feeding. However, there is a lack of information in the literature that could inform a time-dependent model. Also, the quasi-steady state model serves as a first approximation of fine scale drag forces in the precibarium and cibarium.
3. Flow is laminar. This is justified in the next section.

Laminar Flow Assumption

The laminar flow assumption is supported using the equation for calculating the Reynolds number of flow through a cylindrical pipe.

|  | $Re = \frac{\rho VD}{\mu}$ | (1) |
| --- | --- | --- |

Re = Reynolds number

V = average velocity in the pipe

D = diameter of the pipe

µ = dynamic viscosity

The average velocity can be calculated using the volumetric flowrate (Q) and the diameter of the pipe.

|  | $V=\frac{Q}{\pi{(\frac{D}{2})}^{2}}$ | (2) |
| --- | --- | --- |

Thus, the Reynolds number can be calculated using the volumetric flowrate instead of average velocity.

|  | $Re=\frac{4\rho Q}{\pi D\mu}$ | (3) |
| --- | --- | --- |

The Reynolds number increases with decreasing diameter. The thinnest location in the 3D model of the insect precibarium and cibarium that fluid flows through quickly is identified in Figure S11.

**Figure S11.** Location in the 3D model of the precibarium and cibarium with the highest calculated Reynolds number, indicated by a red freeform shape.

A section of the precibarium is shown, with the red freeform shape identifying a region between the precibarial valve and the hypopharyngeal precibarium wall.

The region indicated in Figure S11 is more than 2 µm wide, so the Reynolds number is calculated using a 2 µm diameter.

$$Re=\frac{4(1000\frac{kg}{m^{3}})(4.8*{10}^{-10}\frac{m^{3}}{s})}{\pi(2*{10}^{-6}m)(8.9*{10}^{-4}\frac{kg}{m*s})}=343$$

This Reynolds number is an overestimate since not all the xylem sap passes through the space indicated in Figure S11. Also, a Reynolds number of 343 is far less than the commonly used 2100 - 2300 limit for the laminar flow assumption in cylindrical pipes. Still, the way the precibarial valve protrudes into the lumen of the 3D model suggests that a Kármán vortex street could be formed during egestion. This could cause fluctuating drag forces distal to the precibarial valve. Therefore, care should be taken when interpreting the results of fluid dynamic simulations distal to the precibarial valve. However, the laminar flow assumption is useful for estimating the order of magnitude of drag forces in the insect fluid dynamic simulations.

## S3.3 Calculated Drag Forces

### S3.3a Summary

De La Fuente et al. [1] used microfluidic chamber experiments in a laboratory to determine the flowrates required to detach individually surface-attached *X. fastidiosa* from a glass surface. Then they used fluid dynamic simulations to calculate drag forces acting on bacteria as a function of these flowrates. We recreated their microfluidic chamber simulations to determine the fluid velocities and shear rates that correlated with their reported drag forces. Thus, we were able to correlate flow properties with drag forces and *X. fastidiosa* detachment. We used these correlations to calculate hypothetical drag forces in our xylem and insect simulations, as well as make inferences about *X. fastidiosa* detachment.

### S3.3b Illustrated and Detailed Methods

COMSOL Multiphysics^®^ can be used to calculate fluid drag forces acting on individually surface-attached bacteria using the expression “spf.T_stressx” (Figure S12) [33].


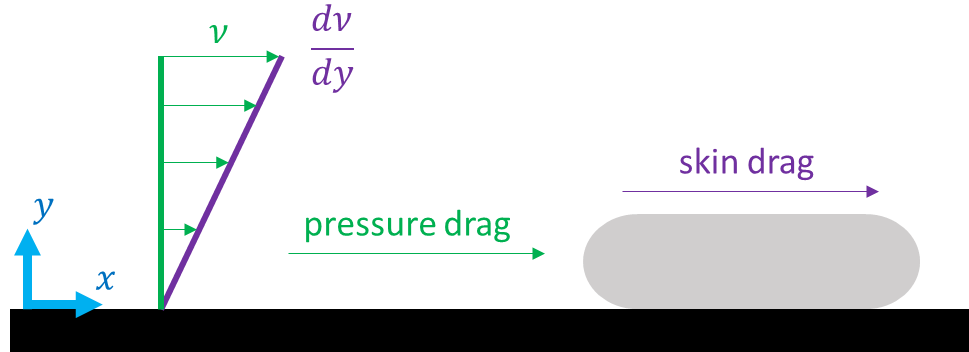


**Figure S12.** Basic diagram of fluid flowing past a surface-attached bacterium (gray).

The fluid has velocities (*ν*) that are variable with respect to distance from the wall surface (*y*). The fluid also has a shear rate (*dν/dy*) that is effectively independent of distance from the wall within a separation distance of 1 µm (Table S2). The bacterium experiences both pressure drag (green) and skin drag (purple). The total drag force can be calculated using the expression “spf.T_stressx” in COMSOL Multiphysics^®^ [33]. The bacterium in this figure is oriented parallel to the flow for the sake of simplicity, but in our microfluidic simulations, the bacterium is oriented perpendicular to flow, as shown in Figure S15.

However, this method of calculating drag forces would be impractical for both the xylem simulations and the insect simulations. It would require an impractical amount of simulations where 3D pill-shaped bacteria would be included at various locations in xylem vessels, the precibarium, and the cibarium. Instead of using this time-consuming method, properties of fluid flow were used to calculate the drag forces that would act on surface-attached bacteria if they were present (Figure S13).


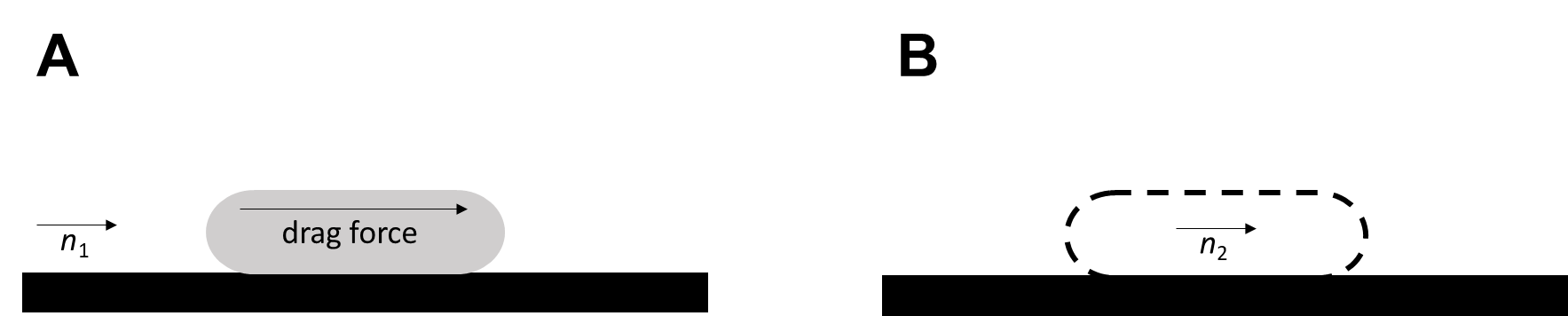


Figure S13. Comparison of the methods for calculating drag forces in our microfluidic chamber simulations vs. in our xylem and insect simulations.

In our microfluidic chamber simulations (A), drag forces acting on the individual surface-attached bacterium were calculated using the expression “spf.T_stressx”. These drag forces were correlated with up stream flow properties (*n*_1_: fluid velocities and shear rates). In our xylem and insect simulations, drag forces acting on hypothetical surface-attached bacteria (dashed oval) are predicted using the flow properties at the locations of the hypothetical bacteria (B) (*n*_2_: fluid velocities and shear rates). The bacteria in this figure are oriented parallel to the flow for the sake of simplicity, but in our microfluidic simulations, the bacterium is oriented perpendicular to flow, as shown in Figure S15.

Drag forces acting on the surface-attached bacteria are composed of both pressure drag (form drag) and skin drag (friction drag), as shown in Figure S12. Pressure drag is a function of local velocity squared and skin drag is a function of local shear rate (Equations 4, 5). Equation 4 uses the dynamic pressure term of Bernoulli’s Equation and Equation 5 is derived from Newton’s law of viscosity.

|  | $F_{pressure\_drag}=\frac{1}{2}\rho A\nu^{2}$ | (4) |
| --- | --- | --- |
|  | $F_{skin\_drag}=\mu A\frac{d\nu}{dy}$ | (5) |

*F* = drag force acting on a surface-attached bacterium

*ρ* = density of xylem sap

*A* = area of the bacteria over which pressure drag or skin drag acts

*ν* = velocity of xylem sap

*µ* = dynamic viscosity of xylem sap

*dν/dy* = shear rate of xylem sap

Previous studies of bacteria detachment have focused on skin drag when calculating fluid dynamic effects on bacteria [7, 34]. This is convenient because calculations are simpler, but our results indicate pressure drag is comparable in magnitude to skin drag at the edge of the precibarial valve during egestion (Figure S14).


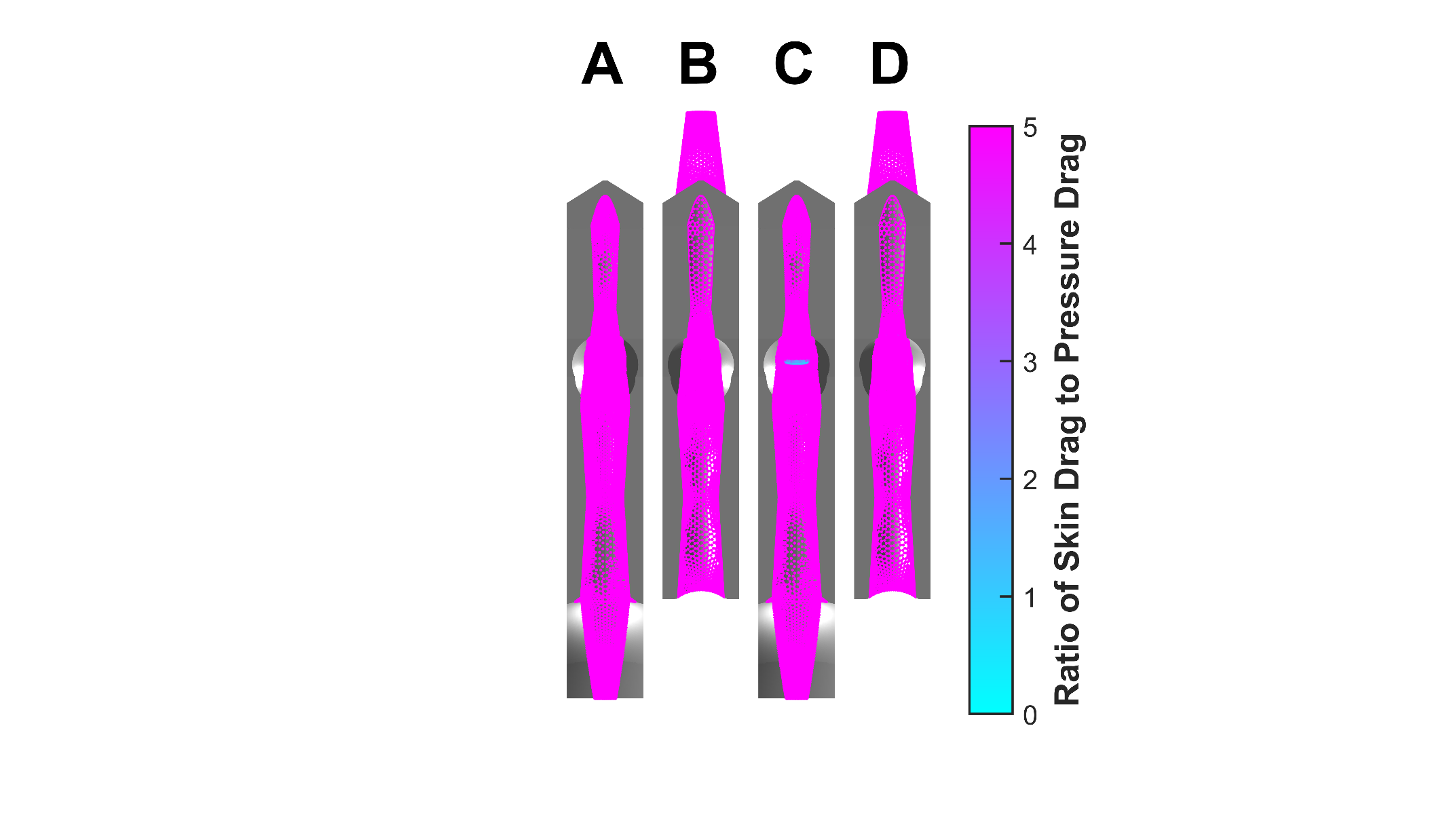


**Figure S14**. Evaluation of the need for including pressure drag in total drag force calculations.

The ratio between skin drag and pressure drag is plotted within gray 3D models of sections of the epipharynx (A & C) and hypopharynx (B & D), which are similar to previously described 3D models [9]. The ratio is generally greater than 5 for both ingestion (A & B) and egestion (C and D), so pressure drag does not have a large effect on total drag force calculations. The ratio is less than 5 at the edge of the precibarial valve during egestion (C), so pressure drag accounts for a relevant portion of the total calculated drag in this region. Pressure drag and skin drag calculations are based on the first and second terms in the fitted equation of Figure S16, respectively.

Fittingly, our drag force calculations for the xylem and insect simulations account for both skin drag and pressure drag, with calculations calibrated based on the work of De La Fuente et al.[1]. We recreated their microfluidic chamber simulations and our results are within 3% of theirs (Figure S15).


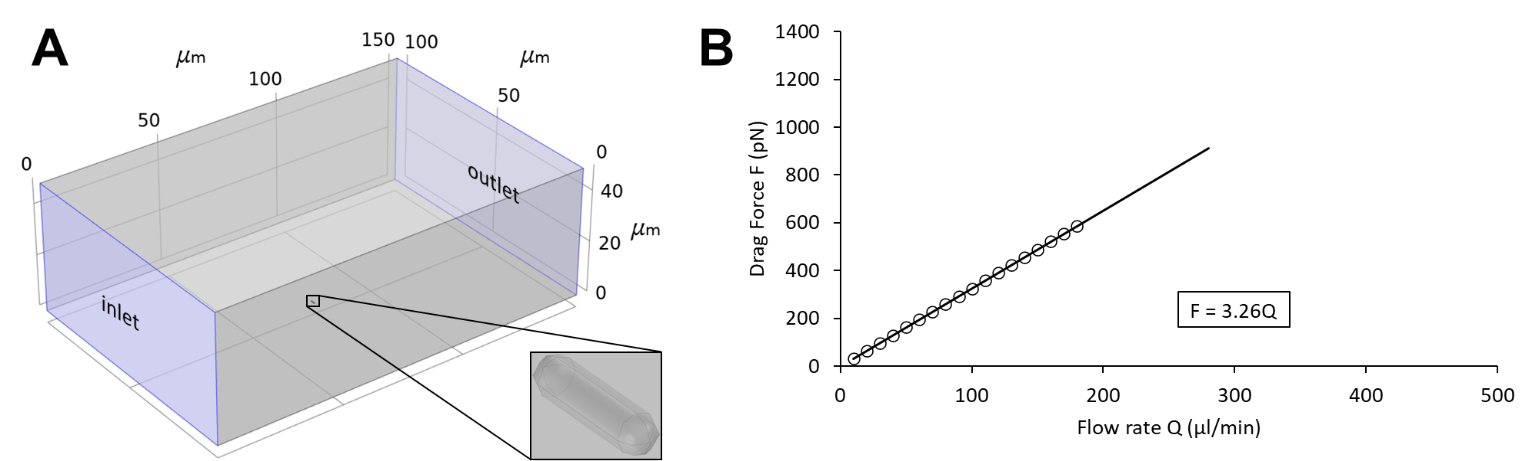


**Figure S15.** Recreation of the results of De La Fuente et al.[1].

A) The microfluidic chamber fluid dynamic simulation was recreated. It calculates the drag force acting on a surface-attached bacterium at different flow rates. The dimensions of the box (150X100X50 µm), inlet/outlet (blue), and bacterium (0.5X1.9 µm) mimic those of De La Fuente et al.[1]. B) Correlation between drag force (F) and flow rate (Q). Our results are within 3% of the results of De La Fuente et al. based on their equation “F = 3.17Q”[1]. Our results were closest to theirs when the bacteria was oriented perpendicular to the flow (A), so this is the orientation used in our microfluidic chamber simulation.

Our microfluidic chamber simulations were probed for the velocities and shear rates up-stream of the surface-attached bacterium (Figure S15). Correlating upstream flow properties with drag forces allowed us to make predictions of drag forces in xylem and insect simulations without including bacteria in the 3D geometries (Figure S15B). We were able to estimate answers to this question:

|  | What drag force would a surface-attached bacterium experience if it were at a specific location in the simulation? |  |
| --- | --- | --- |

Each of the colored dots in Figure S9 and Figure 2 represent an answer to this question. Figure S16 and Equation 6 show the correlation between flow properties and drag force.


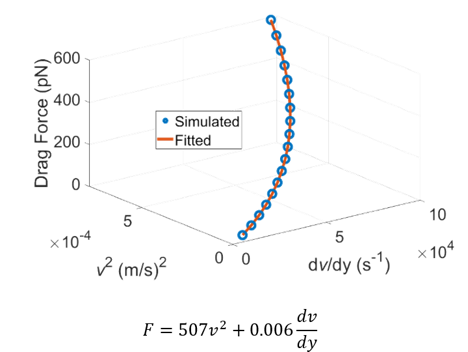


### Figure S16. Correlation between flow properties (*ν^2^* and *dν/dy*) and drag force (*F*) in our microfluidic chamber simulations.

The orange fitted line goes through the blue data point circles, demonstrating a goodness of fit. The equation for the orange fitted line (using SI units) is F = 506.6621985*ν*^2^ + 0.00604035*dν/dy*. This equation is given with rounded coefficients in Equation 6 and the variables are defined below.

|  | $F=507v^{2}+0.00604\frac{dv}{dy}$ | (6) |
| --- | --- | --- |

*F* = total drag force (N)

*ν* = velocity of xylem sap at 0.2887 µm from wall surface (m/s)

*dν/dy* = shear rate of xylem sap at 0.2887 µm from wall surface (s^-1^)

Equation 6 relies on probing the velocities and shear rates 0.2887 µm away from the wall surface where the bacterium is hypothetically attached. This distance from the wall was chosen because it allows for a good fit between the data and model in Figure S16 (better than at 0.25 µm or 0.30 µm). It is preferred to probe velocities at this distance from the wall because pressure drag is dependent on squared velocity. Since the bacterium in the simulation is 0.5 µm wide, the average squared velocity is at 0.2887 µm for the xylem simulations and 0.2852 µm for the insect simulations. These numbers are within 2% of each other and are derived below.

Pressure drag force per area ($\hat{F}$) is dependent on squared velocity:

|  | $\hat{F}=\frac{F_{pressure\_drag}}{A}=\frac{1}{2}\rho\nu^{2}$ | (7) |
| --- | --- | --- |

$F_{pressure\_drag}$= pressure drag force from Equation 4

*A* = area of the bacteria over which pressure drag acts

*ρ* = density of xylem sap

*ν* = velocity of xylem sap

$\hat{F}$ = pressure drag force per area; this term simplifies the calculation because it avoids addressing the position dependency of *A* (the area per nanometer increases from the wall [*y* = 0 µm] to the midline of the bacteria [*y* = 0.25 µm] and then decreases to the outer edge of the bacteria [*y* = 0.5 µm]); the validity of this simplification is supported by the fact that 78% of the area per nanometer over which pressure drag acts is not variable with respect to distance from the wall.

Velocity is dependent on distance from the wall and the slip length[35]:

|  | $\nu=C_{1}(y+l)$ | (8) |
| --- | --- | --- |

*y* = distance from the wall

*l* = slip length specified for a particular simulation

*C*_1_ = shear rate of xylem sap, represented by the letter ‘*C*’ to emphasize its constant nature with respect to distance from the wall in this context (see Table S4).

Plugging Equation 8 into Equation 7:

|  | $\hat{F}=\frac{1}{2}\rho C_{1}^{2}{(y+l)}^{2}$ | (9) |
| --- | --- | --- |

Gathering all constants into a new constant (C_2_):

|  | $\hat{F}=C_{2}{(y+l)}^{2}$ | (10) |
| --- | --- | --- |

Calculating the average pressure drag force per area ($\breve{F}$):

|  | $\breve{F}=\frac{\int_{0}^{y_{2}} \hat{F}dy}{y_{2}}=\frac{C_{2}}{y_{2}}\int_{0}^{y_{2}} {(y+l)}^{2}dy$ | (11) |
| --- | --- | --- |

$y_{2}$ = distance from the wall to the outer edge of the bacteria (0.5 µm)

Integrating:

|  | $\breve{F}=\frac{C_{2}}{y_{2}}\left[ \frac{{(y+l)}^{3}}{3} \right]_{0}^{y_{2}}$ | (12) |
| --- | --- | --- |

Evaluating:

|  | $\breve{F}=\frac{C_{2}}{{3y}_{2}}\left[ {(y_{2}+l)}^{3}-l^{3} \right]$ | (13) |
| --- | --- | --- |

Substituting an expression for $\breve{F}$ based on Equation 10:

|  | $C_{2}{(\breve{y}+l)}^{2}=\frac{C_{2}}{{3y}_{2}}\left[ {(y_{2}+l)}^{3}-l^{3} \right]$ | (14) |
| --- | --- | --- |

$\breve{y}$ = distance from the wall with the average pressure drag force per area

Solving for $\breve{y}$:

|  | $\breve{y}=\sqrt{\frac{{(y_{2}+l)}^{3}-l^{3}}{3y_{2}}}-l$ | (15) |
| --- | --- | --- |

Solving Equation 15 for the xylem simulations:

$$\breve{y}_{xylem}=\sqrt{\frac{{(0.5+0)}^{3}-0^{3}}{3(0.5)}}-0=0.2887 \mu m$$

Solving Equation 15 for the insect simulations:

$$\breve{y}_{insect}=\sqrt{\frac{{(0.5+0.028)}^{3}-{0.028}^{3}}{3(0.5)}}-0.028=0.2852 \mu m$$

An insightful reader might question why the squared velocity profile is central to deciding where fluid properties should be probed, instead of deciding based on shear rate. This question would be reasonable, since skin drag (dependent on shear rate) is generally much larger than pressure drag (dependent on squared velocity) in Figure S14. However, our microfluidic chamber simulations indicate shear rate is effectively independent of distance from the wall within a separation distance of 1 µm (Table S4), so calculated skin drag is effectively independent of probe distance from the wall within this range.

### Table S4. Shear rates (s^-1^) in microfluidic chamber simulations.

Shear rates were probed upstream of the surface-attached bacterium at different distances from the wall. This table shows that sheer rate is effectively independent of distance from the wall within a separation distance of 1 µm.

| **Flow rate** | **Distance from Wall (µm)** | | | | | | | |
| --- | --- | --- | --- | --- | --- | --- | --- | --- |
| **(µl/min)** | **0.01** | **0.1** | **0.2** | **0.25** | **0.3** | **0.5** | **0.75** | **1** |
| **10** | 5387 | 5387 | 5387 | 5387 | 5387 | 5276 | 5277 | 5277 |
| **20** | 10775 | 10775 | 10775 | 10775 | 10775 | 10552 | 10554 | 10555 |
| **30** | 16163 | 16163 | 16163 | 16163 | 16163 | 15830 | 15832 | 15834 |
| **40** | 21552 | 21552 | 21552 | 21552 | 21552 | 21107 | 21110 | 21113 |
| **50** | 26941 | 26941 | 26941 | 26941 | 26941 | 26386 | 26389 | 26393 |
| **60** | 32331 | 32331 | 32331 | 32331 | 32331 | 31665 | 31669 | 31673 |
| **70** | 37721 | 37721 | 37721 | 37721 | 37722 | 36944 | 36949 | 36954 |
| **80** | 43111 | 43112 | 43112 | 43112 | 43112 | 42224 | 42230 | 42236 |
| **90** | 48502 | 48502 | 48503 | 48503 | 48503 | 47504 | 47511 | 47518 |
| **100** | 53893 | 53894 | 53894 | 53894 | 53894 | 52785 | 52792 | 52800 |
| **110** | 59285 | 59285 | 59285 | 59286 | 59286 | 58066 | 58074 | 58082 |
| **120** | 64676 | 64677 | 64677 | 64677 | 64678 | 63348 | 63357 | 63366 |
| **130** | 70069 | 70069 | 70069 | 70070 | 70070 | 68630 | 68639 | 68649 |
| **140** | 75461 | 75461 | 75462 | 75462 | 75462 | 73912 | 73923 | 73933 |
| **150** | 80854 | 80854 | 80855 | 80855 | 80855 | 79195 | 79206 | 79218 |
| **160** | 86247 | 86247 | 86248 | 86248 | 86248 | 84478 | 84491 | 84503 |
| **170** | 91640 | 91641 | 91641 | 91642 | 91642 | 89762 | 89775 | 89788 |
| **180** | 97034 | 97034 | 97035 | 97035 | 97036 | 95046 | 95060 | 95074 |

Equation 7 is not intended to be a rigorous method for quantifying drag forces acting on surface-attached bacteria. It does not account for the different orientations that bacteria may have with respect to flow direction. However, it provides a means of correlating fluid velocities and shear rates with the detachment of *X. fastidiosa* reported by De La Fuente et al [1].

Water Hammer

The water hammer calculation is based on the fundamental equation of water hammer [36]:

|  | $\Delta P=\rho a\Delta\nu$ | (16) |
| --- | --- | --- |

$\Delta P$ = magnitude of pressure wave, based on an instantaneous valve closure

$\rho$ = density of xylem sap

$a$ = speed of sound in xylem sap

$\Delta\nu$ = average velocity of xylem sap (volumetric flow rate divided by cross sectional area)

The density and speed of sound are taken as those of water. The volumetric flow rate is taken as that for ingestion. The cross-sectional area is calculated from a cross-section of the 3D model of the precibarium (Figure S17). Therefore, the maximum pressure spike expected during insect ingestion due to water hammer is overestimated (instantaneous valve closure) as follows:

$$\Delta P=\left( 997\frac{kg}{m^{3}} \right)\left( 1481\frac{m}{s} \right)\left( \frac{3.37\times{10}^{-11}\frac{m^{3}}{s}}{1.14\times{10}^{-10}m^{2}} \right)=4.36\times{10}^{5}Pa=4.3 atm$$

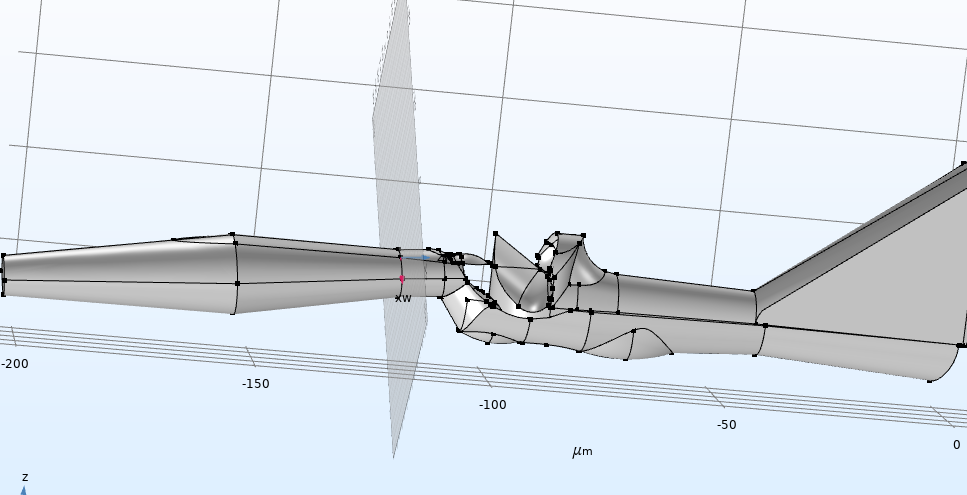


### Figure S17. Cross-section of the precibarium used for water hammer calculation.

A work plane is shown intersecting the 3D model of the precibarium in COMSOL Multiphysics^®^. The cross-section represents the area of the precibarial valve that contacts the fluid distal to the valve when the valve is closed.

# References

1. De La Fuente L, Montanes E, Meng Y, Li Y, Burr TJ, Hoch H, et al. Assessing adhesion forces of type I and type IV pili of Xylella fastidiosa bacteria by use of a microfluidic flow chamber. Appl Environ Microbiol. 2007;73(8):2690-6.

2. Backus EA, Holmes WJ, Schreiber F, Reardon BJ, Walker GP. Sharpshooter X wave: correlation of an electrical penetration graph waveform with xylem penetration supports a hypothesized mechanism for *Xylella fastidiosa* inoculation. Annals of the Entomological Society of America. 2009;102(5):847-67.

3. Backus EA, Shih H-T. Review of the EPG waveforms of sharpshooters and spittlebugs including their biological meanings in relation to transmission of *Xylella fastidiosa* (Xanthomonadales: Xanthomonadaceae). Journal of Insect Science. 2020;20(4):6.

4. Backus EA, Shugart HJ, Gutierrez J, Ebert TA, Walker MA. Field-collected glassy-winged sharpshooters (Hemiptera: Cicadellidae) perform more *Xylella fastidiosa*-inoculating behaviors on susceptible *Vitis vinifera* cv. ‘Chardonnay’ than on resistant *Vitis champinii* grapevines. Journal of Economomic Entomology. 2021;114:1991-2008.

5. Carniello V, Peterson BW, van der Mei HC, Busscher HJ. Physico-chemistry from initial bacterial adhesion to surface-programmed biofilm growth. Advances in colloid and interface science. 2018.

6. Cervantes FA, Backus EA. EPG waveform library for *Graphocephala atropunctata* (Hemiptera: Cicadellidae): effect of adhesive, input resistor, and voltage levels on waveform appearance and stylet probing behaviors. Journal of insect physiology. 2018;109:21-40.

7. Boks NP, Norde W, van der Mei HC, Busscher HJ. Forces involved in bacterial adhesion to hydrophilic and hydrophobic surfaces. Microbiology. 2008;154(10):3122-33.

8. Lovisolo C, Schubert A. Effects of water stress on vessel size and xylem hydraulic conductivity in *Vitis vinifera* L. Journal of Experimental Botany. 1998;49(321):693-700.

9. White D, Backus EA, Marcus IM, Walker SL, Roper MC. Functional foregut anatomy of the blue–green sharpshooter illustrated using a 3D model. Scientific Reports. 2021;11(1).

10. Ranieri E, Zitti G, Riolo P, Isidoro N, Ruschioni S, Brocchini M, et al. Fluid dynamics in the functional foregut of xylem-sap feeding insects: a comparative study of two *Xylella fastidiosa* vectors. Journal of Insect Physiology. 2020;120:103995.

11. Holbrook NM, Zwieniecki MA. Vascular Transport in Plants: Elsevier; 2011.

12. Fine RA, Millero FJ. Compressibility of water as a function of temperature and pressure. The Journal of Chemical Physics. 1973;59(10):5529-36. doi: 10.1063/1.1679903.

13. Elimelech M, Gregory J, Jia X. Particle deposition and aggregation: measurement, modelling and simulation: Butterworth-Heinemann; 2013.

14. Liu C, Li Z. On the validity of the Navier-Stokes equations for nanoscale liquid flows: The role of channel size. AIP Advances. 2011;1(3):032108. doi: 10.1063/1.3621858.

15. Backus EA, McLean DL. The sensory systems and feeding behavior of leafhoppers. I. The aster leafhopper, *Macrosteles fascifrons* Stål (Homoptera, Cicadellidae). Journal of Morphology. 1982;172(3):361-79.

16. Bess E. Blue-green sharpshooter (*Graphocephala atropunctata*) (Signoret, 1854) [Image]. 2012 [updated June 9, 2020]. Available from: https://www.forestryimages.org/browse/detail.cfm?imgnum=5495281.

17. Brodbeck BV, Andersen PC, Oden S, Mizell RF. Preference-performance linkage of the xylem feeding leafhopper, *Homalodisca vitripennis* (Hemiptera Cicadellidae). Environmental entomology. 2014;36(6):1512-22.

18. Stevenson R. Body size and limits to the daily range of body temperature in terrestrial ectotherms. The American Naturalist. 1985;125(1):102-17.

19. Kimball B, Bernacchi C. Evapotranspiration, canopy temperature, and plant water relations. Managed Ecosystems and CO_2_: Springer; 2006. p. 311-24.

20. Reicosky D, Deaton D, Parsons J. Canopy air temperatures and evapotranspiration from irrigated and stressed soybeans. Agricultural meteorology. 1980;21(1):21-35.

21. Do F, Rocheteau A. Influence of natural temperature gradients on measurements of xylem sap flow with thermal dissipation probes. 1. Field observations and possible remedies. Tree physiology. 2002;22(9):641-8.

22. Andersen PC, Brodbeck BV, Mizell III RF. Feeding by the leafhopper, *Homalodisca coagulata*, in relation to xylem fluid chemistry and tension. Journal of Insect Physiology. 1992;38(8):611-22.

23. Son Y, Groves RL, Daane KM, Morgan DJ, Johnson MW. Influences of temperature on Homalodisca vitripennis (Hemiptera: Cicadellidae) survival under various feeding conditions. Environmental entomology. 2009;38(5):1485-95.

24. Son Y, Groves RL, Daane KM, Morgan DJ, Krugner R, Johnson MW. Estimation of feeding threshold for *Homalodisca vitripennis* (Hemiptera: Cicadellidae) and its application to prediction of overwintering mortality. Environmental entomology. 2010;39(4):1264-75.

25. Choi C-H, Westin KJA, Breuer KS. Apparent slip flows in hydrophilic and hydrophobic microchannels. Physics of fluids. 2003;15(10):2897-902.

26. Rapicavoli JN, Kinsinger N, Perring TM, Backus EA, Shugart HJ, Walker S, et al. O-antigen modulates insect vector acquisition of the bacterial plant pathogen, *Xylella fastidiosa*. Applied and environmental microbiology. 2015:AEM. 02383-15.

27. Purcell AH, Finlay A. Evidence for noncirculative transmission of Pierce’s disease bacterium by sharpshooter leafhoppers. Phytopathology. 1979;69(4):393-5.

28. Almeida RP, Purcell AH. Patterns of *Xylella fastidiosa* colonization on the precibarium of sharpshooter vectors relative to transmission to plants. Annals of the Entomological Society of America. 2006;99(5):884-90.

29. Dellieu L, Sarrazin M, Simonis P, Deparis O, Vigneron JP. A two-in-one superhydrophobic and anti-reflective nanodevice in the grey cicada *Cicada orni* (Hemiptera). Journal of Applied Physics. 2014;116(2):024701.

30. Holdgate M. The wetting of insect cuticles by water. Journal of Experimental Biology. 1955;32(3):591-617.

31. Chen L, Zhao X, Pan Y. Establishment of a Standard Method for Boundary Slip Measurement on Smooth Surfaces Based on AFM. Applied Sciences. 2019;9(7):1453.

32. Janssen D, De Palma R, Verlaak S, Heremans P, Dehaen W. Static solvent contact angle measurements, surface free energy and wettability determination of various self-assembled monolayers on silicon dioxide. Thin Solid Films. 2006;515(4):1433-8.

33. Lyu P. How do I compute lift and drag? comsol.com2015 [cited 2020 June 17]. Available from: https://www.comsol.com/blogs/how-do-i-compute-lift-and-drag/.

34. Owens NF, Gingell D, Rutter PR. Inhibition of cell adhesion by a synthetic polymer adsorbed to glass shown under defined hydrodynamic stress. Journal of cell science. 1987;87(5):667-75.

35. Jiménez Bolaños S, Vernescu B. Derivation of the Navier slip and slip length for viscous flows over a rough boundary. Physics of Fluids. 2017;29(5):057103.

36. Ghidaoui MS, Zhao M, McInnis DA, Axworthy DH. A review of water hammer theory and practice. Appl Mech Rev. 2005;58(1):49-76.

1. See Figure S5.13 for calculation method. [↑](#footnote-ref-1)
